# Thermal proteome profiling reveals *Haemonchus* orphan protein HCO_011565 as a target of the nematocidal small molecule UMW-868

**DOI:** 10.3389/fphar.2022.1014804

**Published:** 2022-10-14

**Authors:** Aya C. Taki, Tao Wang, Nghi N. Nguyen, Ching-Seng Ang, Michael G. Leeming, Shuai Nie, Joseph J. Byrne, Neil D. Young, Yuanting Zheng, Guangxu Ma, Pasi K. Korhonen, Anson V. Koehler, Nicholas A. Williamson, Andreas Hofmann, Bill C. H. Chang, Cécile Häberli, Jennifer Keiser, Abdul Jabbar, Brad E. Sleebs, Robin B. Gasser

**Affiliations:** ^1^ Department of Veterinary Biosciences, Faculty of Veterinary and Agricultural Sciences, Melbourne Veterinary School, The University of Melbourne, Parkville, VIC, Australia; ^2^ Walter and Eliza Hall Institute of Medical Research, Parkville, VIC, Australia; ^3^ Melbourne Mass Spectrometry and Proteomics Facility, The Bio21 Molecular Science and Biotechnology Institute, The University of Melbourne, Parkville, VIC, Australia; ^4^ Zhejiang Provincial Key Laboratory of Preventive Veterinary Medicine, College of Animal Sciences, Institute of Preventive Veterinary Medicine, Zhejiang University, Hangzhou, Zhejiang, China; ^5^ Medical Parasitology and Infection Biology, Swiss Tropical and Public Health Institute, Allschwil, Switzerland; ^6^ University of Basel, Basel, Switzerland; ^7^ Department of Medical Biology, The University of Melbourne, Parkville, VIC, Australia

**Keywords:** thermal proteome profiling, anthelmintic discovery, target identification, structure modelling, in silico docking

## Abstract

Parasitic roundworms (nematodes) cause destructive diseases, and immense suffering in humans and other animals around the world. The control of these parasites relies heavily on anthelmintic therapy, but treatment failures and resistance to these drugs are widespread. As efforts to develop vaccines against parasitic nematodes have been largely unsuccessful, there is an increased focus on discovering new anthelmintic entities to combat drug resistant worms. Here, we employed thermal proteome profiling (TPP) to explore hit pharmacology and to support optimisation of a hit compound (UMW-868), identified in a high-throughput whole-worm, phenotypic screen. Using advanced structural prediction and docking tools, we inferred an entirely novel, parasite-specific target (HCO_011565) of this anthelmintic small molecule in the highly pathogenic, blood-feeding barber’s pole worm, and in other socioeconomically important parasitic nematodes. The “hit-to-target” workflow constructed here provides a unique prospect of accelerating the simultaneous discovery of novel anthelmintics and associated parasite-specific targets.

## 1 Introduction

Parasitic diseases of humans and other animals represent a major socioeconomic burden worldwide. In humans, parasitic worms (= helminths) that cause neglected tropical diseases (NTDs) affect ∼1 billion people, equating to a burden of two million disability-adjusted life years (DALYs) (Loukas et al., 2021). In animals, although challenging to quantitate, the disease impact of parasitic helminths is substantial (Pedersen and Fenton, 2015; Charlier et al., 2020; Selzer and Epe, 2020), and some species are also transmissible to humans (i.e., zoonotic) (McCarthy and Moore, 2000; Gordon et al., 2016). The control of these diseases (= helminthiases) relies on diagnosis, treatment and management strategies, and anthelmintic treatment is usually a central component of control campaigns, as vaccines are not available for the majority of parasites, including hookworms (*Ancylostoma* and *Necator*), threadworms (*Strongyloides*) and whipworms (*Trichuris*) (Jex et al., 2011). Anthelmintic treatments are not always highly efficacious/effective, and the reliance on them, particularly if they are used excessively in an uncontrolled (suppressive) manner, has led to the emergence and spread of anthelmintic resistance in parasitic worms (Kotze and Prichard, 2016; Hodgkinson et al., 2019), particularly those of animals. Although the resistance status of worms of humans to available compounds is somewhat unclear (Prichard, 2005; Keiser and Utzinger, 2008; Vercruysse et al., 2011), this is not the case for worms of important production animals (e.g., sheep, goats and cattle), where such resistance is widespread and essentially worldwide.

As developing vaccines against most helminths (particularly nematodes) has been extremely challenging, the focus has been on the discovery and development of new, highly efficacious anthelmintics, intended to circumvent these resistance problems. However, the last new compounds (derquantel and monepantel) were released commercially in 2010, and since that time, no new chemotypes have entered clinical trials. To support discovery efforts, a number of academic teams around the world have contributed to early phase work to identify new anthelmintic entities for subsequent optimisation and development in public-private partnerships (PPPs) (Ramamoorthi et al., 2014; Partridge et al., 2018; Pasche et al., 2018; Taylor et al., 2019; Weber et al., 2019; Clare et al., 2019; HELP (Helminth Elimination Platform), 2019; Taki et al., 2021a).

Our focus has been on screening a range of curated compound libraries using phenotypic assays for the parasitic nematode *Haemonchus contortus*—called the barber’s pole worm (Jiao et al., 2020; Herath et al., 2021; Herath et al., 2022)*.* We employ this worm species, because 1) it is a highly significant, pathogenic nematode with a high reproductive index, and can be readily maintained in a laboratory environment; 2) it represents one of the largest groups (clade V) of socioeconomically relevant nematodes of animals, including a range of species parasitic in humans; 3) it is relatively closely related to *Caenorhabditis elegans* (also clade V)—a free-living nematode—which is one of the best understood multicellular organisms (Holden-Dye and Walker, 2014; Hahnel et al., 2020) and for which extensive biological, biochemical, molecular, genetic and genomic resources exist (Harris et al., 2019; Davis et al., 2022); and 4) it now assumes ‘model organism’ status (Doyle et al., 2020) due to the availability of extensive genomic, transcriptomic, proteomic and lipidomic data sets and resources for this parasitic nematode (Laing et al., 2013; Schwarz et al., 2013; Gasser et al., 2016; Ma et al., 2018; Wang et al., 2018; Wang et al., 2019a; Wang et al., 2019b; Ma et al., 2020a; Wang et al., 2020a; Ma et al., 2020b; Wang et al., 2020b; Doyle et al., 2020; Wang et al., 2021; Wang and Gasser, 2021). Thus, *H. contortus* is exceptionally well suited as a biological tool for anthelmintic discovery.

In recent work, we established a high-throughput whole-organism, phenotypic assay for the screening of relatively large libraries of tens to hundreds of thousands of compounds, the subsequent evaluation of active compounds and structure-activity relationship (SAR) studies to strive toward the optimisation of the activity and potency of analogs (Taki et al., 2021a; Taki et al., 2021b; Shanley et al., 2022). A challenge has been target-deconvolution to support optimisation efforts. Published evidence (Savitski et al., 2014; Perrin et al., 2020; Mateus et al., 2022) demonstrates clearly the exquisite capacity of thermal proteome profiling (TPP) to define or infer target molecules in projects with a biomedical focus (e.g., cancers), but this approach has not yet been utilised to define anthelmintic targets. This context provides the very exciting prospect of being able to rapidly provide evidence of drug-target interactions. Here, we used a high-throughput whole-organism, phenotypic screen of a well-curated compound library to identify new starting points for optimisation against *H. contortus*, and employed TPP to better understand hit pharmacology and support optimisation.

## 2 Results

### 2.1 High throughput screen and potency evaluation identify a drug-like compound (UMW-868) with activity against *H. contortus*

**FIGURE 1:**
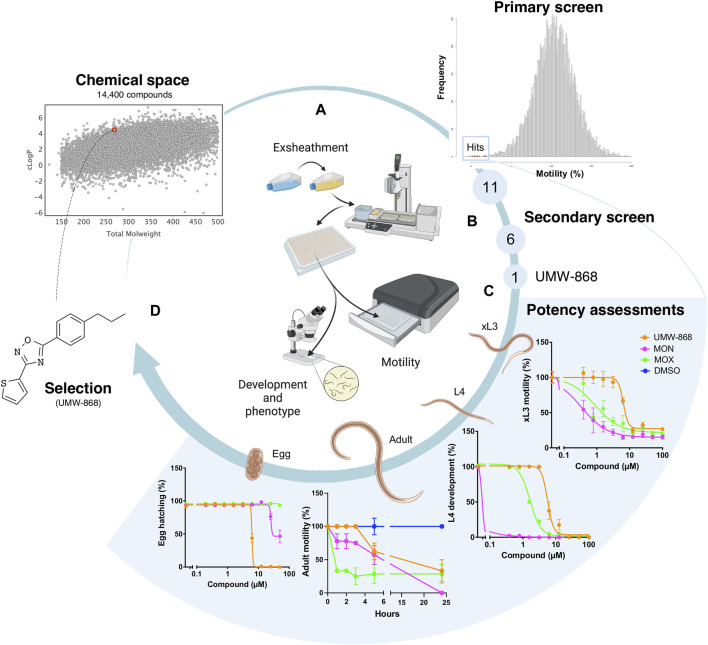
Workflow used for the selection of the candidate nematocidal compound UMW-868. **(A)** All compounds (diverse chemical space) in the HitFinder library (Maybridge) library were screened in an established whole-organism phenotypic assay (Taki et al., 2021a) using exsheathed third-stage (xL3) larvae of *Haemonchus contortus.* The screen of this library identified 11 definitive hits with favourable drug-like features. **(B)** Secondary screen identified six compounds with strong activity, one of which (designated UMW-868) had favourable structural characteristics for further investigation. **(C)** Potency assessments of UMW-868 on different developmental stages of *H. contortus—*i.e., xL3 (motility), L4 (= fourth larval stage; development), female adult (motility) and egg (hatching). **(D)** UMW-868 is linked to the chemical space of the HitFinder library, based on the physicochemical properties (molecular weight and cLogP).

We screened the 14,400 compounds from the Hitfinder Collection from Maybridge (Figure 1A) on exsheathed third-stage larvae (xL3s) of *H. contortus* at a concentration of 20 µM. At 90 h, we identified 36 compounds that reduced xL3 motility by ≥ 70%, equating to an overall “hit rate” of 0.25% (Figure 1A; Supplementary Table S1). At 168 h, 21 of these 36 compounds induced abnormal (non-wildtype) larval phenotypes, detectable with reference to the wildtype (negative control). Next, we selected 11 of the 36 hits (Figure 1A) exhibiting favourable, drug-like features for further evaluations. Dose-response evaluations of motility reduction on xL3s (90 h) and larval development inhibition (168 h) identified 6 of the 11 compounds (Figure 1B) with strong activity with IC_50_ values between 2.6 µM and 11.2 µM. Of these six compounds, UMW-868 was one of the most potent compounds against xL3s of *H. contortus*, has a drug-like structure similar to that of tioxazafen—described as being a “broad-spectrum” nematocide (South and Wilson, 2016) and was, thus, considered as suitable for further assessment (Figure 1C). UMW-868 showed IC_50_ values of 5.6 µM (motility) and 5.8 µM (development), with an ability to induce abnormal (*curved* or *evisceration*) phenotypes in affected xL3s (Figure 1C and Supplementary Figure S1). Subsequently, we assessed the effect of UMW-868 *in vitro* on adult females of *H. contortus* collected directly from the abomasum of a sheep with a patent (30 days) infection. The motility of UMW-868-treated adult females reduced by 37.5% after 5 h of exposure, and by 66.7% after 24 h; the latter reduction was comparable to the moxidectin positive-control (Figure 1C and Supplementary Table S2). The subsequent *in vitro* activity/potency assessment of UMW-868 on the hatching of *H. contortus* eggs revealed an IC_50_ value of 6.2 µM (Figure 1C). Based on the results from potency assessments, UMW-868 was selected for further investigation (Figure 1D).

### 2.2 UMW-868 causes major cell death in *H. contortus*, but no cytotoxicity or mitotoxicity in mammalian hepatic cells *in vitro*

**FIGURE 2:**
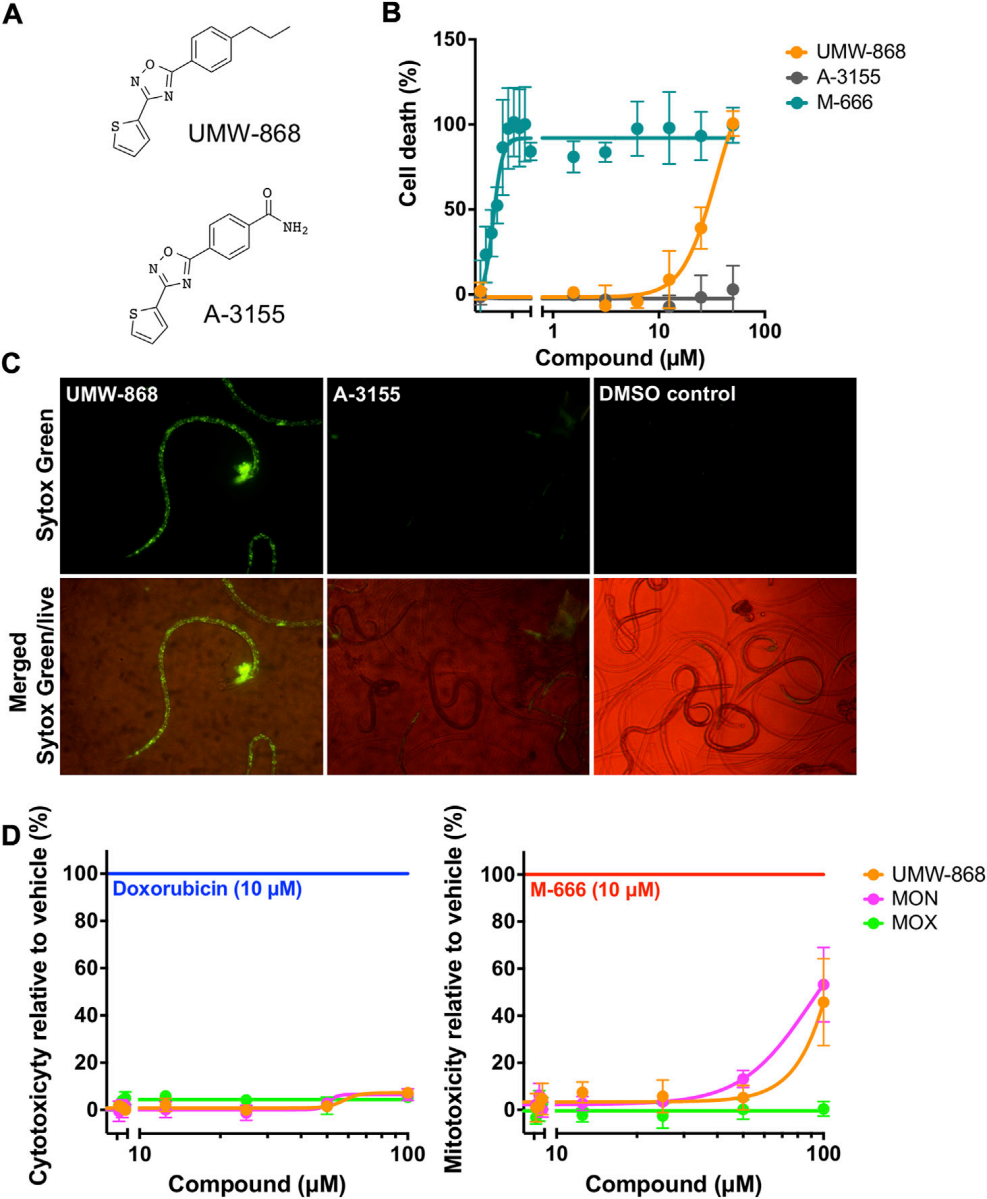
Bioassay characterisation of compound UMW-868. **(A)** Structures of UMW-868 (active) and its analog A-3155 (inactive). **(B)** Dose-response assessment of the *in vitro* lethality of UMW-868 for inducing cell death in exsheathed third-stage larvae (xL3s) of *Haemonchus contortus* over 90 h, with reference to xL3s exposed to control compounds, M-666 (lethal) and A-3155 (non-lethal). **(C)** Dead cells detected microscopically of UMW-868-treated xL3s stained with Sytox Green nucleic acid stain, observed under 40-times magnification, with reference to xL3s exposed to A-3155 (non-lethal) and no-compound control (containing 0.2% (*v/v*) DMSO). **(D)** Cellular and mitochondrial toxicities of UMW-868 and two non-toxic compounds, monepantel (MON) and moxidectin (MOX) on HepG2 human hepatoma cells, using control compounds (doxorubicin and M-666) for respective toxicities.

Initially, we measured cell death in UMW-868-treated xL3s of *H. contortus*. After 90 h of exposure, cell death was detected in the UMW-868-treated xL3s (>5 µM), while xL3s treated with an inactive analog (A-3155) remained viable (Figure 2A). UMW-868 killed 100% of treated xL3s at 50 μM, with an IC_50_ value of 34.8 µM (Figure 2B). After 168 h of exposure to UMW-868, dead cells could be seen throughout the larvae (Figure 2C), while no dead cells were detected in xL3s exposed to A-3155 or DMSO (Figure 2C). Next, we tested UMW-868 on HepG2 cells and mitochondria for toxicity; UMW-868 did not display any appreciable cytotoxicity or mitotoxicity at concentrations of up to 100 µM (Figure 2D).

### 2.3 Activity of UMW-868 on other nematodes *in vitro* and *in vivo*

We investigated the activity of UMW-868 on other species of parasitic nematodes of humans and animals, and on the free-living nematode *Caenorhabditis elegans* (Supplementary Table S3). After 72 h of exposure at 10 µM *in vitro*, UMW-868 killed ∼100% of third-stage larvae (L3s) and adults of *Ancylostoma ceylanicum* and of L3s of *Strongyloides ratti*. At 1 μM, UMW-868 killed 50% of *A. ceylanicum* adults and 30% of *S. ratti* L3s, but did not affect *A. ceylanicum* larvae at this concentration. UMW-868 was also partially effective at killing other species of nematodes including *Necator americanus* (13%), *Heligmosomoides polygyrus* (13%) and *Trichuris muris* (24%). After 72 h of exposure at 10 µM *in vitro*, UMW-868 reduced the motility of *C. elegans* by 53.5% at 40 h, and had an IC_50_ value of 9.9 µM. Subsequently, we assessed the activity of UMW-868 against *He. polygyrus* in mice and *A. ceylanicum* in hamsters (Supplementary Table S4). Using a single oral dose of 100 or 200 mg/kg body weight, UMW-868 reduced the intensities of *A. ceylanicum* and *He. polygyrus* infections by 48% and 47%, respectively. Throughout these experiments, there was neither evidence of acute toxicity in animals, nor was there evidence of pathological changes on skin or in internal organs (liver, lung, spleen and gastrointestinal tract) upon post-mortem examination of animals.

### 2.4 Evidence that orphan protein HCO_011565 is a target of UMW-868

**FIGURE 3:**
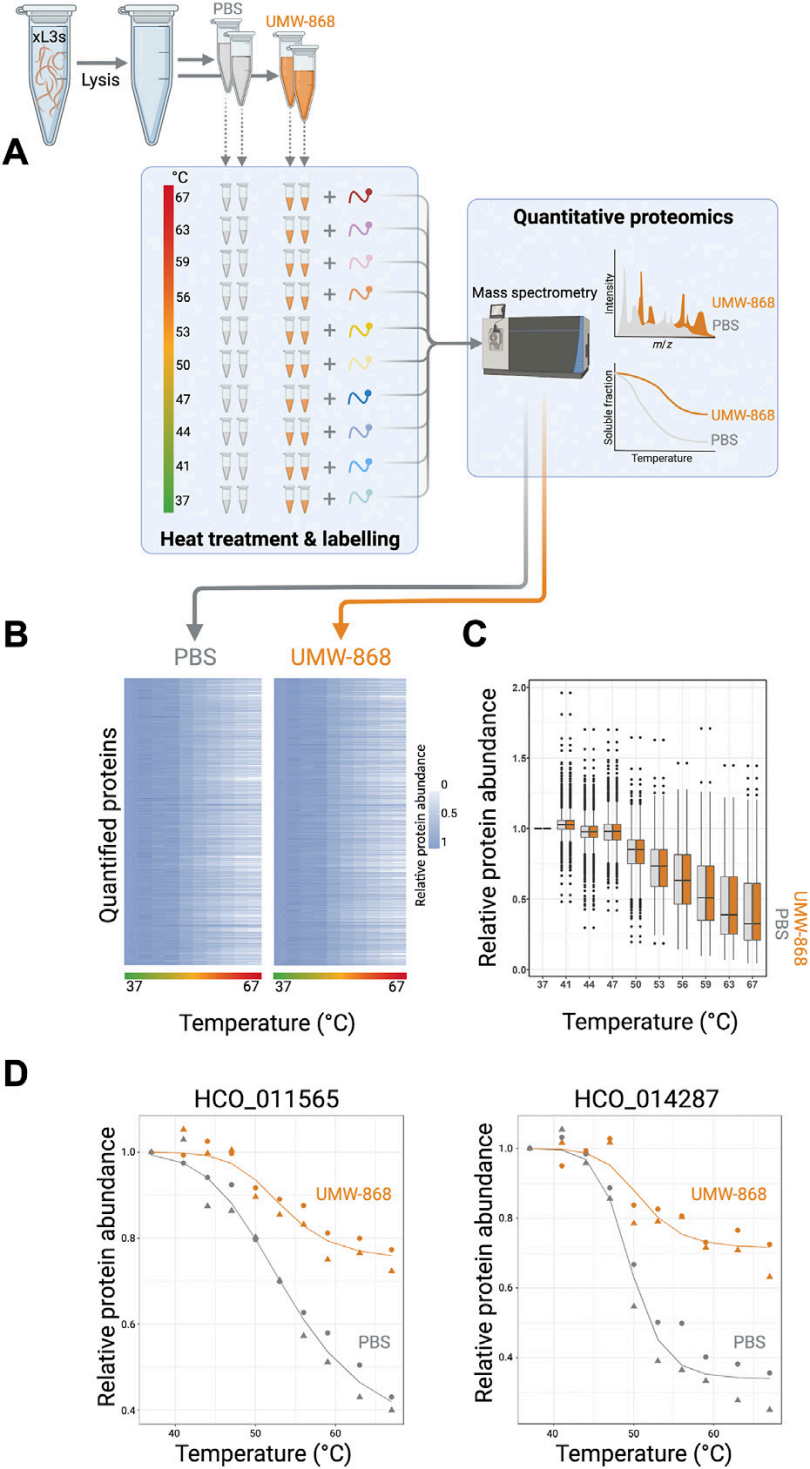
Thermal proteome profiling. **(A)** Proteins from exsheathed third-stage larvae (xL3s) of *Haemonchus contortus* were released by cell lysis in phosphate-buffered saline (PBS) containing 0.4% NP-40 (detergent). Prior to heat treatment, aliquots were incubated with UMW-868 or PBS, respectively. Subsequently, remaining soluble proteins were collected by centrifugation and analysed by tandem mass tag (TMT)-based quantitative proteomics. Melting curves of each quantified protein were plotted using the R package. **(B)** Heat map of quantified soluble proteins showing relative protein abundances upon treatment with UMW-868 or PBS with temperature gradient from 37°C to 67°C. Normalised protein abundance is shown as a grey to white scale, depicting high to low relative protein abundance. **(C)** Boxplot of overall quantified soluble proteins showing relative fold-change in abundance upon treatment with UMW-868 or PBS in a temperature gradient from 37°C to 67°C. **(D)** Thermal shift plot showing the melting curves of two potential protein targets for UMW-868: HCO_011565 and HCO_014287. Data from two replicates.

Given the clear evidence that UMW-868 has anthelmintic activity and no cell or mitochondrial toxicity in mammalian hepatic cells, we aimed to identify this compound’s target/s. Using thermal proteome profiling (TPP), we identified and quantified a total of 3,678 *H. contortus* proteins (Figure 3A; Supplementary Table S5), a subset of the somatic proteome defined recently for this parasite (Wang et al., 2019b). The full list of quantified proteins at each temperature point along the gradient (37–67°C) is given in Supplementary Table S4. Both heatmap and box plot analyses revealed marked decreases in relative protein abundances for UMW-868- or PBS-treated *H. contortus* xL3-homogenate samples with increasing temperature (Figures 3B,C). After assessing thermal profiles of quantified proteins by nonparametric analysis of response curves (NPARC), and fitting nonparametric models to the temperature profile data under the null and alternative hypotheses (Childs et al., 2019), we yielded 2,591 distinct melting curve profiles. Following filtering, we identified 55 proteins whose thermostability and abundance were altered in the presence of UMW-868. Of these 55 proteins, two uncharacterised (unknown or orphan) proteins (encoded by genes HCO_011565 and HCO_014287, respectively) exhibited the highest, statistically significant thermostability in the presence of UMW-868 with reference to the phosphate-buffered saline (PBS) control (Figure 3D), suggesting that they are targets for UMW-868.

**FIGURE 4:**
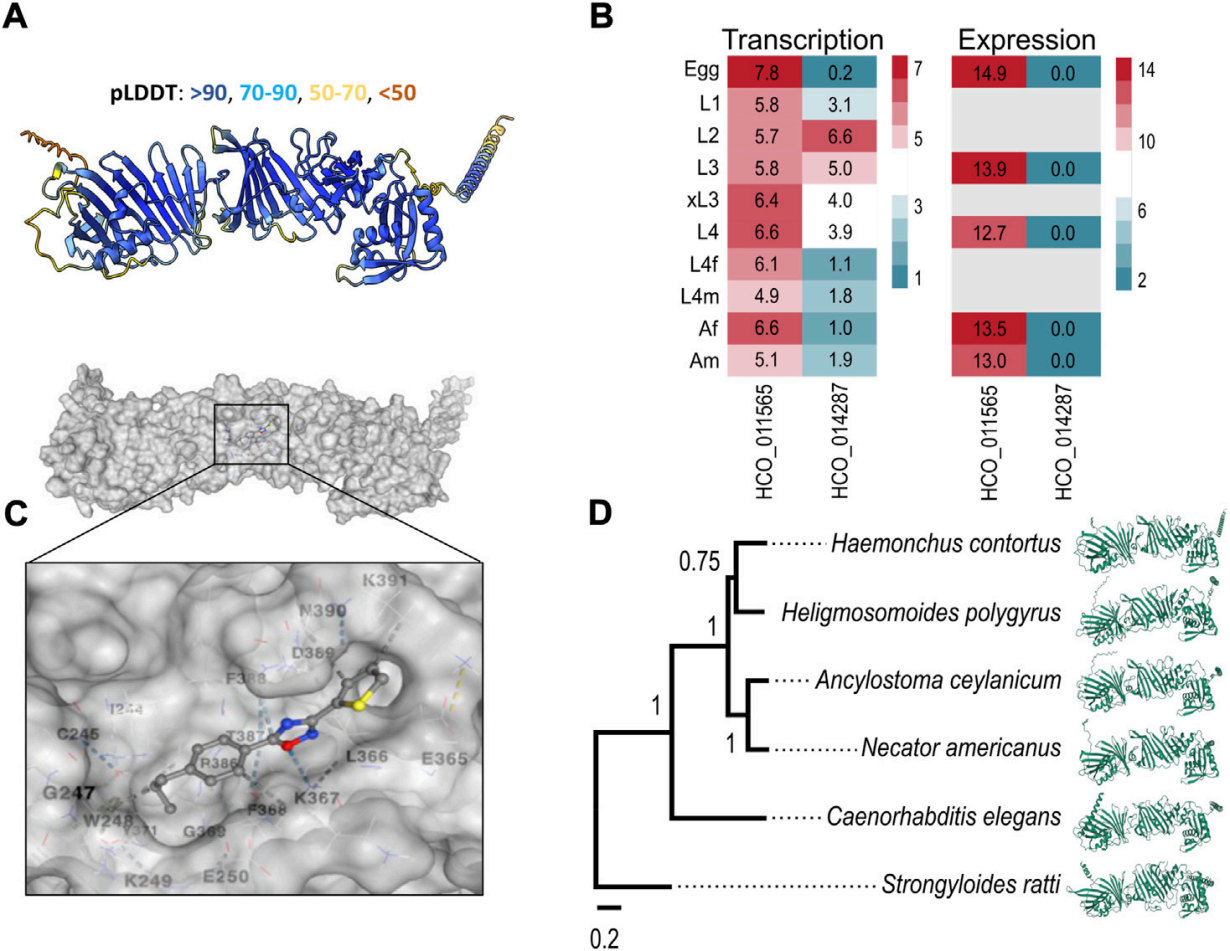
Molecular characterisation of HCO_011565 and docking with UMW-868. **(A)** Structure of HCO_011565 protein predicted *in silico* using the Alphafold2 algorithm. The per-residue confidence scores (pLDDT; left to right) for individual sections of the protein are indicated. **(B)** Transcription and expression profiles for the gene encoding HCO_011565 in different developmental stages/sexes of *Haemonchus contortus* larvae: eggs, first-, second- and third-stage larvae (L1, L2 and L3, respectively), exsheathed third-stage larvae (xL3s), fourth-stage larvae (L4s), female and male L4s (L4f and L4m, respectively), and female and male adults (Af and Am, respectively). Heatmaps indicate levels of transcription/expression: red indicates moderate to high transcription/expression; green is low expression; and grey indicates that expression was not detected. **(C)** Three-dimensional structure of the protein HCO_011565 and its interaction with compound UMW-868; the inset shows the positioning of UMW-868 in the pocket predicted within HCO_011565. **(D)** Phylogenetic relationship of protein HCO_011565 with its orthologs in five other species of nematode (left) and the structural models of these orthologs predicted using Alphafold (right).

In the absence of crystal structures for these two proteins, we predicted their conformations using a high-performance algorithm, AlphaFold2 (Jumper et al., 2021) (cf. Materials and Methods; Section 4). Although AlphaFold could not predict a confident structure for HCO_014287, the model for HCO_011565, containing one transmembrane domain at the C-terminus, was well-supported (confidence score of 87.3; Figure 4A). Subsequently, we explored transcription/expression levels for these two molecules. For HCO_011565, we showed a constitutive transcription in all key developmental stages of *H. contortus* and a high expression in the egg, L3, L4 (fourth larval) and adult stages. For HCO_014287, we recorded moderate to high transcription only in the L2 (second larval) and L3 stages, but limited or no expression in other developmental stages (Figure 4B). Based on these findings, HCO_011565 was selected for further investigation.

*In silico* docking experiments indicated that UMW-868 docked into a pocket (cavity size: 384 Å^3^) of HCO_011565 with a Vina score of -6.9 (Figure 4C). Given that UMW-868 was shown to have distinct levels (13–100%) of anthelmintic activities *in vitro* or *in vivo* against distinct developmental stages of nematodes other than *H. contortus*, including *A. ceylanicum, C. elegans, He. polygyrus*, *N. americanus* and *S. ratti*, we studied the primary protein sequences of HCO_011565 orthologs in these nematodes, modelled individual proteins and then compared the structural models (Figure 4D). Despite significant/marked amino acid differences (19.9%–67.7%) among primary sequences, respective predicted structures were relatively conserved (confidence scores of 85.4–87.3), upon pairwise comparison (Supplementary Figure S2). UMW-868 was also predicted to dock into a pocket in the *C. elegans* ortholog (Y92H12BR.3a) of HCO_011565, with a Vina score of −6.3 (Supplementary Table S6). However, this was not the case for HCO_011565 orthologs of the other nematode species studied, suggesting that UMW-868 has one or more alternative targets in these worms. Although the differences in binding modes predicted here might partially explain the variable activities/potencies of UMW-868 recorded for individual nematode species studied, other factors, such as developmental stage, presence/absence of a cuticular sheath (i.e., L3s *versus* other stages) and ability to ingest/uptake and absorb/transport the compound; nature and extent target protein expression in a particular developmental stage; and/or type of assay employed (i.e., *in vitro* or *in vivo*), also need careful consideration.

### 2.5 Medicinal chemistry leads to enhanced potency in *H. contortus*

**FIGURE 5:**
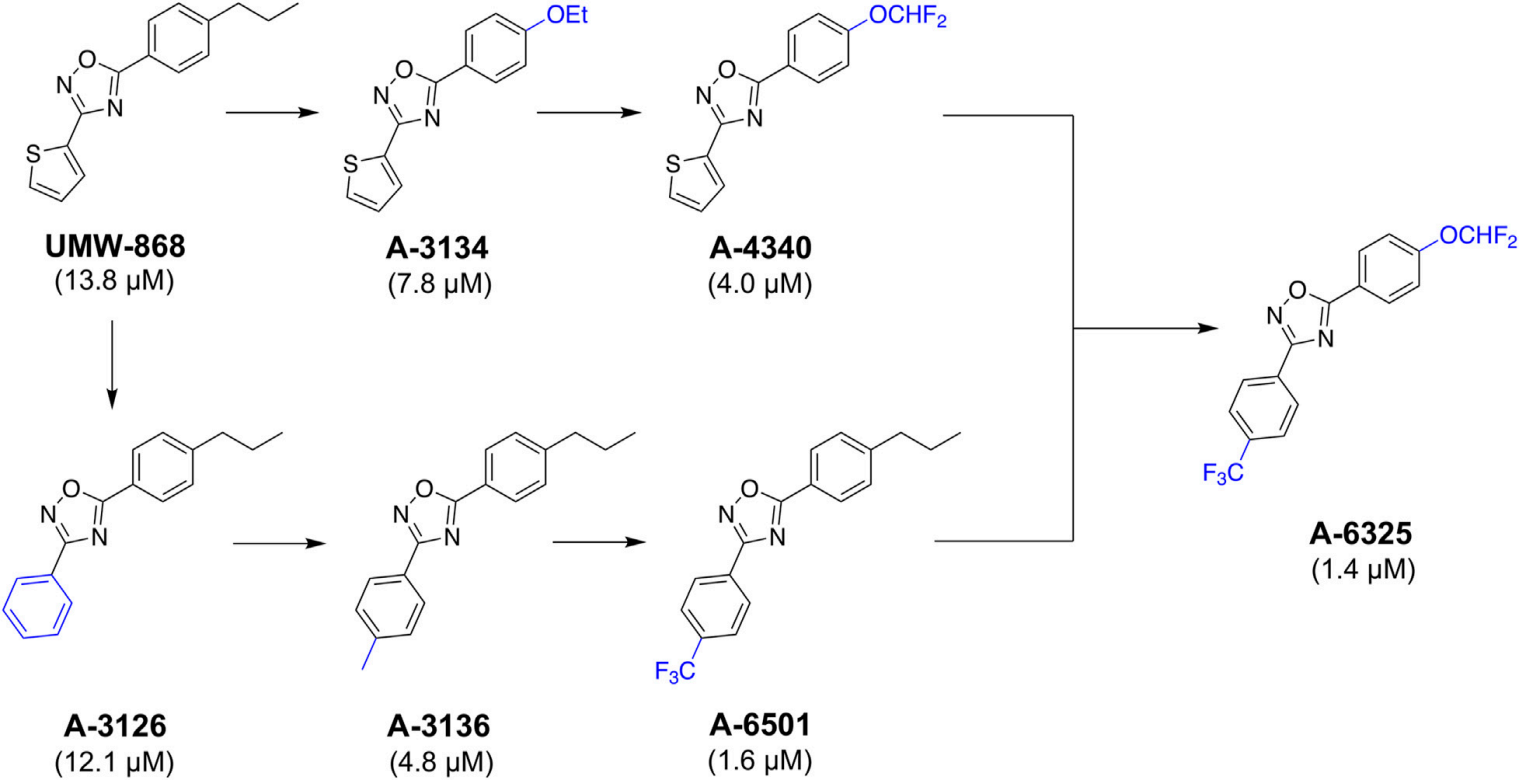
UMW-868 and its analogs evaluated for activity on *Haemonchus contortus*. Sequential modifications of UMW-868 analogs with substitutions of the aryl ring (bottom) and *n*-propyl (top). Potencies (IC_50_ in µM) of individual compounds on the motility of exsheathed third-stage larvae (xL3s) of *H. contortus* are indicated.

Given UMW-868's promising activity and structure as a nematocidal candidate, we focused on modifying its chemistry (Figure 5) to attempt to enhance potency and further assessed cyto- and mito-toxicity on HepG2 cells (Table 1). In the “southern region” (*R* ^*1*^) of UMW-868, the substitution of aryl ring with thiophene did not alter potency to reduce xL3-motility (A-3126; IC_50_ 12.1 µM), as expected. However, the potency increased with the addition of 4-methyl to aryl group (A-3136; IC_50_ 4.8 µM), and enhanced further with 4-trifluromethyl substitution (A-6501; IC_50_ 1.6 µM). In the “northern region” (*R* ^*2*^), the replacement of the *n*-propyl substituent with an ethoxy group increased potency (A-3134; IC_50_ 7.8 µM), and was enhanced further through the introduction of a 4-difluromethoxy group (A-4340; IC_50_ 4.0 µM). In accordance with the results for xL3-motility reduction, some of these modified analogs (i.e., A-3136, A-6318, A-6501 and A-4340) had enhanced inhibitory activity/potency on larval development (Table 1). Interestingly, the related nematocidal compound—tioxazafen (A-8417) (South and Wilson, 2016)—was not active against xL3s, and did not inhibit larval development or egg hatching (IC_50_ > 50 μM; see Table 1). Finally, the combination of the best south (*R* ^*1*^) and north (*R* ^*2*^) modifications, an analog with 4-trifluromethyl substituent on the phenyl ring and a 4-difluromethoxy group replacing the *n*-propyl substituent (i.e., A-6325), gave an IC_50_ value of 1.4 µM for xL3 motility reduction (Table 1). These findings were in accord with trends seen in the Vina scores predicted from *in silico* docking (Supplementary Table S6).

**TABLE 1:** Potencies (IC_50_ in µM) of UMW-868 analogs with iterations of the aryl (*R* ^*1*^) and *n*-propyl (*R* ^*2*^) substituents of UMW-868 and tioxazafen on the motility of exsheathed third-stage larvae (xL3s) and development of fourth-stage larvae (L4s) at 168 h, and the hatching of eggs of *Haemonchus contortus* at 48 h; and results from an assessment of the toxicity of individual compounds on human hepatoma (HepG2) cells (CC_50_) and the mitochondria (MC_50_) of these cells.

Compound	*R* ^*1*^	*R* ^*2*^	*H. contortus*			HepG2 (human hepatoma) cells	
	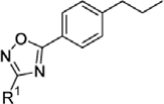	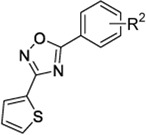	xL3 motility reduction (IC_50_ in µM)	L4 development inhibition (IC_50_ in µM)	Egg hatch inhibition (IC_50_ in µM)	Cytotoxicity (48 h) CC_50_ (μM)	Mitotoxicity (48 h) MC_50_ (μM)
UMW-868	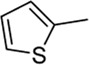	*n*-propyl	13.8	50.0	6.2	> 50.0	> 50.0
A-3126	Ph	—	12.1	50.0	13.1	> 50.0	> 50.0
A-3136	4-MePh	—	4.8	12.5	7.5	> 50.0	> 50.0
A-6501	4-CF_3_Ph	—	1.6	6.3	> 50.0	> 50.0	> 50.0
A-6318^a^	4-OMePh	—	1.4	1.6	38.0	> 50.0	48.2
A-4347^a^	—	H	> 50.0	> 50.0	> 50.0	> 50.0	> 50.0
A-3134	—	4-OEt	7.3	> 50.0	> 50.0	> 50.0	> 50.0
A-4340	—	4-OCHF_2_	4.0	25.0	> 50.0	> 50.0	> 50.0
A-6316^a^	—	4-OCF_3_	9.8	> 50.0	> 50.0	> 50.0	> 50.0
Tioxazafen	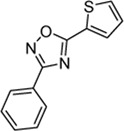		> 50.0	> 50.0	> 50.0	> 50.0	> 50.0	
A-6325	4-CF_3_Ph	4-OCHF_2_	1.4	3.1	> 50.0	> 50.0	36.3

a Compounds included to guide the structure activity relationship to reach A-6325 (Figure 5).

## 3 Discussion

Here, in a high-throughput phenotypic screening assay, we identified a tioxazafen-related anthelmintic candidate (UMW-868) that has reproducible and quite potent nematocidal activity against *H. contortus* and at least three other nematode species. Using thermal proteome profiling (TPP), we discovered a novel protein target (designated HCO_011565) in *H. contortus* that interacts with UMW-868; we inferred a statistically confident structure for HCO_011565 using the most advanced structural prediction program available, AlphaFold2 (Jumper et al., 2021); and we revealed clear structural homology of HCO_011565 with orthologs in other key nematode species, but no homology match to any vertebrate protein in public databases. These findings (proof-of-principle) indicate/suggest that the final workflow established here (Supplementary Figure S4)—which combines *in vitro* screening, potency assessment and TPP-guided protein target identification with *in silico* structural prediction and ligand docking—might be a useful tool to support the discovery of novel anthelmintics and matching “orphan” target candidates for prioritisation and subsequent evaluation. This has major implications, given the widespread problem of anthelmintic resistance in nematodes of major agricultural and veterinary significance worldwide.

The discovery of a previously unknown, parasite-specific protein target candidate, HCO_011565, which has structural homologs in a range of parasitic nematodes, but is absent from host animals, is particularly attractive. Given the consistent expression/transcription of HCO_011565 in key developmental stages of *H. contortus* (Figure 4), and evidence of chemical knock-down and cell death in *H. contortus* caused by UMW-868 (Figures 1, 2; Table 1), we propose that HCO_011565 is an essential molecule—a proposal that will require experimental validation in the laboratory. In our opinion, essential orphan proteins, such as this, which are encoded by single-copy genes, are of major interest, because there is no redundancy (i.e., no paralogs within a species) in the target. Unpublished investigations by our group have estimated that 10–30% of protein-encoding genes encoded in the genomes of *H. contortus* and related parasitic nematodes are currently unknown (and are, thus, assigned orphan status), and have no homologs in any other invertebrate or vertebrate species for which comprehensive genomic or transcriptomic data are presently available in public databases. Although homology has usually been assessed based on primary nucleotide and/or protein sequence comparisons, we now advocate for a structure-based approach, whereby proteins predicted to be orphans are first modelled using the algorithms AlphaFold2 (Jumper et al., 2021) and/or RosETTA (Baek et al., 2021), and then directly compared with structures available in the Protein Data Bank (PDB) using the server Dali (Holm, 2020). Such an approach will lead to a better annotation of orphan proteins, improve comparative analyses, and guide the selection and prioritisation of parasite-specific targets that are absent from host animals and invertebrates in the environment, which is particularly important to consider, given the challenges/problems that may arise following the (commercial) release of a new parasiticide, such as adverse impacts due to toxicity on the environment and on critically important invertebrates, including honey bees, other pollinators and endangered species, and the consumers of food (Beynon, 2012a; Beynon, 2012b; EFSA (European Food Safety Authority), 2019; Rasmussen et al., 2021).

*In silico* investigation indicated that there are structural orthologs of HCO_011565 in other nematodes known to be susceptible to UMW-868 *in vitro* and/or *in vivo* (Figure 4), but such orthologs do not exist in host animals. UMW-868 was inferred to dock specifically into its proposed binding pocket in HCO_011565 (Figure 4C), and into other sites in HCO_011565 orthologs of other nematodes (i.e. *He. polygyrus, A. ceylanicum*, *N. americanus*, *C. elegans* and *S. ratti*; Supplementary Table S6). The evidence from TPP does indicate that UMW-868 binds to HCO_0011565, but we do not exclude the possibility that there might be other targets. Thus, further work would be needed to address this latter issue, and also to validate the proposed binding site (pocket) for UMW-868 in HCO_0011565 and its orthologs in other nematodes. Although the structure of tioxazafen is similar to that of UMW-868, and appears to bind in the same pocket in HCO_011565, it is predicted not to dock as well as UMW-868 does (Vina score: -6.2; Supplementary Table S6), which is consistent with the finding that tioxazafen does not significantly affect the motility, development or viability of *H. contortus* larvae *in vitro* (Table 1), contrasting information presented in an ICT patent (South and Wilson, 2016). While tioxazafen may interact with HCO_011565 (Supplementary Figure S3), it is readily possible that it has no actual target in *H. contortus* larvae. Although other UMW-868 analogs (e.g., A-4347, A-3134, A-4340 and A-6316), particularly those with *n*-propyl substituents, were predicted to dock into, or interact with, the same pocket in protein HCO_011565 (cf. Supplementary Figure S3), they were also inactive against one or more stages of *H. contortus*, suggesting that factors such as mode of uptake, absorption, transport, metabolism and/or biotransformation might explain this lack of activity. Overall, this work suggests that comparative docking might be a useful tool to support the initial selection of anthelmintic-target candidates, prior to future docking by molecular dynamic simulation (MDS) (Śledź and Caflisch, 2018) and crystal structure-based investigations. We now propose that mechanism of action studies of UMW-868 be undertaken in *H. contortus*, for which extensive resources and tools exist for functional and SAR investigations; future work is also needed to verify that HCO_011565 orthologs in other nematodes are genuine targets.

Taken together, the results of this study show that the combined laboratory-computational workflow established here has considerable potential for the discovery of novel anthelmintic targets and parasiticides.

## 4 Materials and methods

### 4.1 Compound library

The HitFinder Collection comprises 14,400 (synthetic) small molecular compounds representing the drug-like diversity of the Maybridge Screening Collection (Thermo Fisher Scientific). These compounds were selected from the collection using a clustering algorithm employing standard Daylight Fingerprints (Daylight Software; www.daylight.com) with the Tanimoto similarity index (Tanimoto, 1958), clustering at 0.71 similarity. The physicochemical properties of compounds, including cLogP and molecular weight, were calculated and analysed using the DataWarrior software (Sander et al., 2015) (version 5.5.0). All compounds in the library fit the five Lipinski rules (Lipinski, 2000) for “drug-likeness,” have a purity of >90% and have been selected to be non-reactive, minimising false positive results and ensuring high-quality results. These compounds were supplied at a concentration of 10 mM in 100% dimethyl sulfoxide (DMSO; Sigma-Aldrich).

### 4.2 Production and storage of *H. contortus*

The Haecon-5 strain of *H. contortus* was maintained in experimental sheep as described previously (Schwarz et al., 2013; Preston et al., 2015), in accordance with the institutional animal ethics guidelines and the regulations of Australia (permit no. 1714374; University of Melbourne). In brief, helminth-free Merino sheep (6 months of age; male) were orally inoculated (*via* a gastric tube) with 7,000 L3s of *H. contortus.*

Four weeks after infection, faecal samples containing *H. contortus* eggs were collected daily from sheep with patent infection. *H. contortus* L3s were produced from eggs by incubating these faecal samples at 27°C and >90% relative humidity for 7 days (Preston et al., 2015), and collected in tap water and sieved through two layers of nylon mesh (pore size: 20 μm; Rowe Scientific) to remove debris or dead larvae, and then stored (at a concentration of 2,000 L3s per ml) at 11°C for up to 6 months (Preston et al., 2015). Immediately prior to use, L3s were exsheathed and sterilised by incubation in 0.15% (*v/v*) bleach at 38°C for 20 min^20^. Following this standard treatment, exsheathed L3s (xL3s) were immediately washed five times in 50 ml of sterile saline by centrifugation at 500 *g* (5 min) at room temperature (22°C–24°C). After the last wash, xL3s were suspended in sterile lysogeny broth (Bertani, 1951), supplemented with 100 IU/ml of penicillin, 100 μg/ml of streptomycin and 0.25 μg/ml of amphotericin B (Fungizone^®^, Thermo Fisher Scientific)—designated LB*.

### 4.3 High throughput screening of compounds on *H. contortus*

An established platform was used for compound screening (Taki et al., 2021a). Using a semi-automated liquid handling robot (VIAFLO ASSIST PLUS, Integra Biosciences), compounds were individually diluted to 20 µM in LB* containing 0.2% (*v/v*) DMSO and then dispensed in 20 µl into the wells of sterile 384-well flat bottom microtitre plates (cat. no. 3680; Corning); 320 compounds were arrayed on each plate, with 16 wells being negative controls (with LB* + 0.2% DMSO) and four wells containing each monepantel (Zolvix; Elanco), moxidectin (Cydectin; Virbac), monepantel + abamectin (Zolvix Plus; Elanco) and compound MIPS-0018666 (abbreviated here as M-666) (Le et al., 2018) as positive controls (20 µM). Following the dilution and dispensing of compounds into plates, 80 *H. contortus* xL3s in 20 μl of LB* were transferred to individual wells; after this step, the final concentrations were 20 µM of test- or positive-control compound, and 0.2% of DMSO. During dispensing, xL3s were maintained in a homogenous suspension using a constant stream of bubbles produced employing an aquarium pump (H2Pro). Plates containing *H. contortus* xL3s were incubated at 38 °C, 10% (*v/v*) CO_2_ and >90% relative humidity.

After 90 h of incubation with compounds (20 μM), *H. contortus* xL3s motility was measured for 15 min in individual wells of each plate by infrared light beam-interference (Simonetta and Golombek, 2007) using a WMicroTracker ONE instrument (Phylumtech). Raw data captured were normalised against measurements obtained for the positive (monepantel) and negative (LB* + 0.2% DMSO) controls to remove plate-to-plate variation by calculating the percentage of motility using the program GraphPad Prism (v.9.1.2; GraphPad Software). A compound was considered active (“hit”) if it reduced larval motility by ≥ 70%. To continually assess the performance of the screening assay, the Z′-factor (Zhang et al., 1999) was calculated (using data for the DMSO and the M-666 controls) for individual plates (*n* = 43) and was consistently ≥0.7; reliable assays achieve a Z′-factor of between 0.5 and 1. The signal to background (S/B) ratio (for the same controls) (Sui and Wu, 2007) was consistently >200.

Following the measurement of xL3 motility, plates were incubated again for 78 h (i.e., a total of 168 h) under the same conditions. Thereafter, 40 µl of 1% (*v/v*) iodine were added to individual wells, and worms examined using a light microscope (M80, Leica) at 60-times magnification to assess larval development, based on the presence/absence of a well-developed pharynx (Sommerville, 1966), and/or phenotypic alterations with respect to untreated xL3s.

### 4.4 Dose-response evaluations on *H. contortus*

These evaluations were carried out to estimate the half-maximal inhibitory concentrations (IC_50_ values) for hit compounds against *H. contortus* xL3s. With reference to the two positive-control compounds (monepantel and moxidectin), IC_50_ values for compounds were estimated (Preston et al., 2015; Taki et al., 2021a) using two-fold serial dilution (in LB*; 9 points from 50 μM to 0.2 µM) in 96-well plates (cat. no. 3596; Corning) with larvae at a density of 300 *H. contortus* xL3s in 100 µl per well. Plates containing *H. contortus* xL3s were incubated at 38°C, 10% (*v/v*) CO_2_ and >90% relative humidity.

The motility of *H. contortus* xL3s and L4 development were measured following 168 h of incubation with compound (Taki et al., 2021a). Compound concentrations were log_10_-transformed and fitted using a variable slope four-parameter equation, constraining the highest value to 100% employing a least squares (ordinary) fit model using GraphPad Prism software (v.9.1.2). Compounds were tested by three independent experiments in triplicate. A one-way analysis of variance (ANOVA) with a Tukey’s multiple comparison test or an unpaired *t*-test was used to establish statistically significant differences in larval motility or development.

### 4.5 Assessing compound activity against the adult and egg stages of *H. contortus*

Adult females of *H. contortus* collected from the abomasum of an infected sheep with patent infection (4 weeks) were used to assess the activity of compound UMW-868 in an established motility assay (Le et al., 2018). Each compound was added in triplicate to the wells of a 24-well plate (cat. no. 3524; Corning) at a concentration of 100 μM in 500 μl of phenol-red free Roswell Park Memorial Institute 1,640 medium (RPMI; Thermo Fisher Scientific) supplemented with 100 IU/ml of penicillin, 100 μg/ml of streptomycin and 0.25 μg/ml of amphotericin B (designated RPMI*). Two positive-control compounds, monepantel and moxidectin, at 100 μM, and medium containing 0.2% (*v/v*) DMSO were included in the same plate in triplicate to serve as negative controls. Four adult females of *H. contortus* were assessed in each of the triplicate wells containing the test compound, and the positive and negative controls, and incubated for 24 h at 38°C, 10% (*v/v*) CO_2_ and >90% relative humidity. At 1, 2, 3, 5 and 24 h during the incubation, a video recording (30 s) of each well was taken to assess the reduction in worm motility, using scores of 3 (“good”), 2 (“low”), 1 (“very low”) or 0 (“no movement”) (Taki et al., 2020). For each test or control compound, the motility scores for each of the triplicate wells were calculated, normalised against the scores of the negative control (100% motility) and recorded as a percentage.

Compounds were also assessed for their effect on the hatching of eggs of *H. contortus in vitro*. To do this, eggs were isolated from the faeces from sheep with patent (30 day-) *H. contortus* infection by sucrose flotation (Mes et al., 2007). Eggs were suspended in H_2_O at a concentration of one egg per µl. The eggs were dispensed into wells (in triplicate; 100 eggs per well) and incubated with two-fold serial dilutions (9 points from 50 μM to 0.2 µM in H_2_O) of individual test or control compounds for 48 h at 27°C and >90% relative humidity; wells with no compound were also included. The numbers (percentages) of eggs from which first-stage larvae (L1s) hatched were counted using a light microscope (M80, Leica) at a 40-times magnification. With reference to positive-control compounds (i.e., monepantel and moxidectin), IC_50_ values of compounds were calculated.

### 4.6 Assessing cell lethality in *H. contortus*

Fluorescence staining was employed to assess the ability of UMW-868 to reduce the viability of *H. contortus.* The xL3s were dispensed in 96-well plates at a density of 300 xL3 per well, and incubated with compounds UMW-868 or A-3155, non-active analogs of UMW-868, at 50 μM at 38°C, 10% (*v/v*) CO_2_ and >90% relative humidity. After 90 h of incubation, *H. contortus* xL3s were stained with Sytox Green nucleic acid stain (Thermo Fisher Scientific) at the final concentration of 1 µM in LB* in the same 96-well plates and incubated for 1 h at 38°C and 10% (*v/v*) CO_2_ with >90% relative humidity. Following the incubation, the viability of larvae was evaluated by measuring the relative fluorescent units (RFU) of xL3s using an Omega FLUOstar microplate reader (BMG Labtech), with excitation and emission wavelengths set at 485 nm and 520 nm, respectively. The 96-well plates containing the stained larvae were returned to the incubator for further 78 h; after a total of 168 h of compound exposure, the stained larvae were examined under an inverted fluorescence microscope (DM IL, Leica) equipped with a green fluorescent protein filter (470/500/525 mm; Leica), and imaged using Leica Application Suite software (Leica).

### 4.7 Testing the cellular and mitochondrial toxicities of compounds in HepG2 cells

The toxicities of compounds on cell and mitochondria were assessed to estimate their half-maximal cytotoxic concentrations (CC_50_) and half-maximal mitotoxic concentrations (MC_50_), respectively. HepG2 human hepatoma cells were seeded into wells of a 96-well plate at a density of 5.5 × 10^4^ cells per well in 80 µl of Dulbecco’s modified eagle medium (DMEM; with GlutaMax™ cat. no. 10566016 or 4 mM L-glutamine cat. no. 11966025; Thermo Fisher Scientific), supplemented with 25 mM D-glucose (cytotoxicity) or D-galactose (mitotoxicity), 10% (*v/v*) inactivated foetal bovine serum (iFBS), 100 IU/ml of penicillin, 100 μg/ml of streptomycin and 0.25 μg/ml of amphotericin B (designated DMEM*). Cells were allowed to adhere for 16 h at 37°C and 5% (*v/v*) CO_2_ at > 90% relative humidity, then, incubated for 48 h with individual, serially-diluted compounds (two-fold serial dilution in DMEM*; seven points from 50 μM to 1.56 µM) in a final volume of 100 µl. For the assessment of toxicity on mitochondria, cells were starved of serum (DMEM* without iFBS) for 4 h prior to the 48 h-incubation with compounds (Swiss and Will, 2011; Kamalian et al., 2015). Two positive control compounds, doxorubicin (cytotoxic control) and M-666 (mitotoxic control) were each added to three individual wells in each 96-well plate at a single concentration of 10 µM. DMEM* + 0.25% (*v/v*) DMSO was added to 12 wells in each plate as negative controls. Monepantel and moxidectin were also serially diluted — prepared in the same manner as test compounds — and used as (“non-toxic”) reference compounds. After 48 h of incubation, cell viability was determined by the crystal violet staining method (Śliwka et al., 2016). The absorbance (595 nm) of treated cells was normalised with reference to the negative control (100% viability). To determine the CC_50_ and MC_50_ values, compound concentrations were log_10_-transformed, baseline-corrected using a respective positive control (doxorubicin or M-666) and fitted using a variable slope four-parameter equation with a least squares (ordinary) fit model using GraphPad Prism software (v.9.1.2). Compounds and controls were tested in triplicate.

### 4.8 Procurement of nematodes other than *H. contortus*

*Ancylostoma ceylanicum* and *Necator americanus* (hookworms) were each routinely maintained in Syrian golden hamsters (three week-old males from Janvier Laboratories, Le Genest-Saint-Isle), *Heligmosomoides polygyrus* in NMRI mice (three week-old females from Charles River; Sulzfeld), *Trichuris muris* in C57BL/6NRj mice (three week-old females from Janvier Laboratories), and *Strongyloides ratti* in Wistar rats (three week-old males from Janvier Laboratories) (Buchter et al., 2021; Keiser and Häberli, 2021), in accordance with the institutional animal ethics guidelines and the regulations of Switzerland (permit no. 2070; Swiss Tropical and Public Health Institute).

Hamsters were orally infected with 140 L3s of *A. ceylanicum* or 150 L3 *N. americanus*, rats were subcutaneously injected with 1,300 L3s of *S. ratti*, and mice were orally inoculated with 88 L3s of *He. polygyrus* or 200 embryonated *T. muris* eggs (Buchter et al., 2021; Keiser and Häberli, 2021). L3s of each *A. ceylanicum*, *N. americanus* and *He. polygyrus* were produced from eggs by incubating faeces from monospecifically infected animals for 8–10 days in the dark at 24°C and >90% humidity, and then isolated and concentrated using the Baermann technique (Garcia, 2001). *A. ceylanicum* and *N. americanus* L3s were suspended in Hanksʼ balanced salt solution (HBSS; Thermo Fisher Scientific), *He. polygyrus* L3s in RPMI 1640 medium and *S. ratti* L3s in phosphate-buffered saline (PBS, pH 7.4), with each medium containing 100 IU/ml of penicillin, 100 IU/ml of streptomycin and ∼0.25 μg/ml of amphotericin B.

Adult *A. ceylanicum* and *S. ratti* were collected from the intestines of infected hamsters and rats after 3–6 weeks of infection. Adult *T. muris* were collected from the intestines of infected mice after 7 weeks of infection. *A. ceylanicum* were suspended in HBSS supplemented with 10% (*v/v*) inactivated foetal calf serum (Bioconcept AG, Allschwil), *S. ratti* RPMI 1640 medium supplemented with 5% (*v/v*) inactivated foetal calf serum and *T. muris* in RPMI 1640 medium plus 5% (*v/v*) inactivated foetal calf serum, with each medium containing penicillin/streptomycin and amphotericin B (same concentrations as for L3s), and incubated at 37 °C in 5% (*v/v*) CO_2_.

### 4.9 Assessing compound activity *in vitro* against other nematodes

For each *A. ceylanicum*, *He. polygyrus* and *S. ratti*, L3s were dispensed into 96-well plates at a density of 30–40 L3s per well, and exposed to 1 µM and/or 10 µM of each compound in a final volume of 250 µl. Each compound was tested in triplicate at each concentration. L3s in media containing 1% (*v/v*) DMSO served as a negative control. Plates were kept in the dark at 22°C, and those containing *N. americanus* was incubated at 37°C, 5% (*v/v*) CO_2_ and >90% relative humidity. After 72 h of incubation with compounds, larval death was assessed by counting motile L3s following stimulation with 50–80 μl of hot water (∼80 °C).

For each *A. ceylanicum*, *S. ratti* and *T. muris*, adult worms (both sexes) were dispensed into wells of 24-well plates at a density of three (*A. ceylanicum* and *T. muris*) or six (*S. ratti*) worms per well, and exposed to 1 μM and 10 µM of compound or DMSO (same concentrations; negative control) in a final volume of 2.0 ml. For each concentration, compounds were tested in duplicate. *A. ceylanicum* and *S. ratti* were incubated at 22°C (in the dark), and *T. muris* at 37°C, 5% (*v/v*) CO_2_ and >90% relative humidity. After a 72 h incubation, adult worms in individual wells were examined microscopically using a viability scale from 3 (“normal activity”) to 0 (“dead”), following stimulation with 500 μl of hot water (∼80°C) (Keiser et al., 2016).

### 4.10 Evaluating compound activity *in vivo* against other parasitic nematodes

Candidate compound(s) were tested for their effect on other nematode species (*He. polygyrus* and *A. ceylanicum*) in infected rodents.

For *He. polygyrus*, four NMRI mice (3 weeks old; female) were infected orally with 88 *He. polygyrus* L3s, and immunosuppressed with 0.25 mg/ml dexamethasone (Sigma-Aldrich), in the drinking water, until 2 days prior to treatment (Mes et al., 2007). For *A. ceylanicum,* four Syrian golden hamsters (3 weeks old; male) were infected orally with 300 L3s without immunosuppression (Tritten et al., 2012). Fourteen days or 23 days after the infection, mice and hamsters, respectively, were treated orally with UMW-868 at dosages of 100 mg/kg *bw* (mice and hamsters). Four each of untreated mice and hamsters served as placebo controls. Six to 7 days post-treatment with the compound, all animals were euthanised by CO_2_ (hamsters being initially narcotised using isoflurane), and the intestine was dissected. The live adult worms were collected and counted to calculate the worm burden reductions and worm expulsion rates. The worm burden reduction was calculated using the mean number of live worms for the treated group and establishing the differences, in a percentage, with reference to the untreated control group.

### 4.11 Thermal proteome profiling

TPP is an advanced, multiplexed mass-spectrometry method that allows the unbiased identification or detection of drug targets (Savitski et al., 2014; Perrin et al., 2020; Mateus et al., 2022); it relies on the thermostability of protein-drug interactions to identify potential target protein(s) through denaturation profiles (melting curves) upon heat treatment (Childs et al., 2019), and has proven capacity to specifically identify targets of anti-cancer drugs or drug candidates (Savitski et al., 2014; Perrin et al., 2020; Mateus et al., 2022). Here, we followed an established TPP protocol (Jafari et al., 2014), which has five steps (i.e., preparation of parasite protein extracts; incubation with compound and “heating” of samples; protein digestion and peptide labelling; mass spectrometric analysis; and data processing and analysis; cf. Figure 3):

(i) Preparation of protein extracts from *H. contortus:* L3s of *H. contortus* were exsheathed using established methods (Taki et al., 2021a), collected by centrifugation (2,000 x *g* for 5 min) and frozen at −80 °C following the removal of the supernatant. Subsequently, the frozen pellet (containing 30,000 xL3s) was ground to a fine powder in liquid nitrogen using a mortar and pestle, transferred to a 10 ml tube, suspended in 3 ml ice-cold phosphate-buffered saline (pH 7.0) containing 0.5% (*v/v*) nonyl phenoxypolyethoxylethanol (NP-40), with or without protease inhibitors (cocktail set I; Merck, Denmark; TPP results achieved with inhibitors were the same as without), and lysed by gentle aspiration/expulsion using a 5 ml sterile syringe with a 22-gauge needle. Subsequently, the supernatant was collected from this suspension following centrifugation at 20,000 x *g* for 20 min at 4°C. The protein concentration in the supernatant was measured using a BCA Protein Assay Kit (Thermo Fisher Scientific), adjusted to 2 mg/ml and divided into four 250 μl aliquots/replicates (each containing 500 μg protein).
(ii) Incubation with compound (UMW-868), and temperature profile: Of the four 250 µl aliquots of xL3 proteins, two (i.e., test-samples) were each incubated with an equal volume of compound (UMW-868 at 50 μM), and two (control-samples) with an equal volume of PBS (pH 7.0) for 30 min at 23°C. Each of the samples (containing 500 µl) was partitioned into 10 PCR tubes (50 µl each); individual pairs of test- and control-samples were simultaneously incubated in a thermal cycler (Applied Biosystems) at 10 distinct temperatures (37, 41, 44, 47, 50, 53, 56, 59, 63 and 67°C) for 3 min. Subsequently, all 40 tubes were centrifuged 20,000 x *g* for 20 min at 4°C, and soluble proteins (i.e., from above the pellet) collected into fresh tubes (each containing 45 µl).
(iii) In-solution digestion and isobaric stable isotope labelling of peptides: Proteins in aliquots (45 µl) of individual samples (*n* = 40) were denatured in 8 M urea for 30 min at 37°C and diluted to <2 M urea using lysis buffer prior to processing for in-solution digestion (Ang et al., 2011). Samples were reduced with 10 mM tris (2-carboxyethyl) phosphine (TCEP), alkylated with 55 mM iodoacetamide, followed by digestion with trypsin (Promega) at 37°C for 16 h. The trypsin-treated samples were acidified with 1.0% (*v/v*) formic acid (FA) and purified using Oasis HLB cartridges (Waters; wash solvent, 0.1% FA; elution solvent, 80% acetonitrile (ACN) in 0.1% FA). Then, proteins were labelled with tandem mass tags (TMTs) (Zecha et al., 2019). In brief, desalted peptides were resuspended in 50 mM triethylammonium bicarbonate (TEAB) (pH 8.5) and mixed with a TMT10plex reagent (Thermo Fisher Scientific) that dissolved in 41 μl anhydrous acetonitrile. The TMT-peptide mixture was incubated for 1 h at 25°C with gentle shaking. Sequentially, 3.2 μl of 5% (*w/v*) hydroxylamine was added to the mixture and incubated for 15 min at 25°C with gentle shaking to quench the reaction. Labelled peptides were combined accordingly and then desalted on Oasis HLB cartridges (Waters; using wash solvent, 0.1% FA; elution solvent, 80% acetonitrile (ACN) in 0.1% FA). Each mixed peptide sample was separated into eight fractions using the high pH reversed-phase peptide fractionation kit (Pierce), according to the manufacturer’s protocol. All fractions were freeze-dried prior to resuspension in aqueous 2% (*w/v*) acetonitrile and 0.05% (*w/v*) trifluoroacetic acid (TFA) before LC-MS/MS analysis.
(iv) LC-MS/MS analysis, and protein identification/annotation: LC-MS/MS was performed on the Exploris 480 Orbitrap mass spectrometer (Thermo Fisher Scientific). The LC system was equipped with an Acclaim Pepmap nano-trap column (Dinoex-C18, 100 Å, 75 μm × 2 cm) and an Acclaim Pepmap RSLC analytical column (Dinoex-C18, 100 Å, 75 μm–50 cm). The tryptic peptides were injected into the enrichment column at an isocratic flow of 5 μl/min of 2% (*v/v*) CH_3_CN containing 0.05% (*v/v*) TFA for 6 min, applied before the enrichment column was switched in-line with the analytical column. The eluents were 0.1% (*v/v*) FA (solvent A) in H_2_O and 100% (*v/v*) CH_3_CN in 0.1% (*v/v*) FA (solvent B), both supplemented with 5% DMSO. The gradient was at 300 nl/min from (i) 0–6 min, 3% B; (ii) 6–7 min, 3%–4% B; (iii) 7–82 min, 4%–25% B; (iv) 82–86 min, 25%–40% B; (v) 86–87 min, 40%–80% B; (vi) 87–90 min, 80–80 3% B; (vii) 90–90 min, 80%–3% B and equilibrated at 3% B for 10 min before injecting the next sample. The Exploris 480 Orbitrap mass spectrometer was operated in the data-dependent mode, whereby full MS1 spectra were acquired in a positive mode, with spray voltage at 1.9 kV, source temperature at 275°C, MS1 at 120,000 resolution, normalised AGC target of 300% and maximum IT time of 25 ms. The top 3 s method was used and selecting peptide ions with charge states of ≥ 2–7 and intensity thresholds of ≥ 5e^3^ were isolated for MS/MS. The isolation window was set at 0.7 m/z, and precursors were fragmented using higher energy C-trap dissociation (HCD) at a normalised collision energy of 35, a resolution of 30,000 (TurboTMT activated), a normalised AGC target of 200% and automated IT time.

Mass spectrometry data were processed using MaxQuant (Tyanova et al., 2016) for the identification and quantification of peptides/proteins. Proteins were matched to those inferred from the reference genome (version 4) for *H. contortus* (Doyle et al., 2020) and sequences in the NCBI non-redundant (nr) database (Pruitt et al., 2012). The MaxQuant default methods were used for reporter MS2 TMT based workflow. The TMT reagent was corrected for natural carbon isotopes and incomplete stable isotope incorporation. Fixed modifications of carbamidomethylation of cysteine. Trypsin/P was set as the protease with a maximum of two missed cleavages. Variable modifications are oxidation of methionine and acetylation of protein N-terminus. Protein and PSM false discovery rates (FDR) were both set at < 0.01. Results are available *via* the PRIDE data repository (accession number: PXD034868).
(v) Data processing and analysis: The protein data produced by MaxQuant was taken for analysis in R (v.4.1.2). Decoy proteins, contaminant proteins, proteins only identified by modified peptides, and proteins that were identified by less than two razor or unique peptides were removed. Corrected reporter ion intensities were then divided by the intensity of the 37°C channel. In two cases, where TMT reporter ion channels showed significantly lower intensity than expected, values were imputed by taking the average of the two adjacent channels. Due to the marked decrease in overall protein abundance with increasing temperature, protein abundance ratios were grouped by treatment temperature and subjected to quantile normalisation using the limma (v3.50.0) (Ritchie et al., 2015). Proteins were filtered to retain only those with non-zero values for each sample, and these were taken for subsequent analysis.
Thermal profiles of quantified proteins were assessed using the package NPARC (version 1.6.0) (Childs et al., 2019), which fits nonparametric models to the temperature profile data under null and alternative hypotheses; *p*-values were then calculated from F-statistics with empirically estimated degrees of freedom, as described in the NPARC package documentation (Perrin et al., 2020). Melting profiles were plotted and manually inspected for top ranking protein hits that were statistically significant (Benjamini-Hochberg-adjusted *p*-values were <0.05).

### 4.12 Protein structure prediction, and protein-ligand docking

The three-dimensional structures of proteins were predicted using the machine learning algorithm AlphaFold2 (Jumper et al., 2021), which is an advanced, artificial intelligence (AI) program—developed by DeepMind and acquired by Alphabet/Google; this program uses a neural network (deep-learning) (Jumper et al., 2021) to predict the three-dimensional structures of proteins from their primary amino acid sequences, and achieves high levels of accuracy/confidence compared with conventional, homology-based modelling methods. Here, to model interactions between *Haemonchus* protein and the compound (UMW-868), the docking server CB-Dock (http://cao.labshare.cn/cb-dock/) was used for automated cavity detection and evaluation (Liu et al., 2019) of selected proteins, followed by performing the protein-ligand docking using AutoDock Vina algorithm; resultant “Vina” scores reflected binding modes (Trott and Olson, 2010). Protein structures were displayed in ChimeraX (Pettersen et al., 2021).

### 4.13 Phylogenetic analysis

The amino acid sequences of orthologous proteins were aligned using MAFFT v7.490 employing the linsi option (Katoh and Standley, 2013). Gaps and poorly aligned regions of the alignment were removed using trimAl v1.4. rev15 using the -automated1 option (Capella-Gutierrez et al., 2009). The Akaike Information Criteria (AIC) test in ModelFinder (Kalyaanamoorthy et al., 2017) selected the LG model of evolution for subsequent phylogenetic analysis (Le and Gascuel, 2008). Bayesian phylogenetic inference (BI) was determined using Markov chain Monte Carlo (MCMC) analysis in MrBayes (Ronquist et al., 2012). One million generations of MCMC analysis were performed, and trees were recorded every 200th generation. At this point, the standard deviation of split frequencies was <0.01, and the potential scale reduction factor (PSRF) approached 1. Consensus trees (50% majority rule) were generated using the final 75% of trees. Trees were annotated and enhanced using the ggtree R package (v1.16.6) (Yu et al., 2017), and nodal support values on trees were indicated as posterior probabilities (pp).

### 4.14 Chemistry procedures

All non-aqueous reactions were performed under an atmosphere of nitrogen, unless otherwise specified. Commercially available reagents were used without further purification. Flash chromatography was performed with silica gel 60 (particle size 0.040–0.063 μm) on a CombiFlash Rf Purification System (Teledyne Isco) with mobile phase gradients as specified. NMR spectra were recorded on a Bruker Avance DRX 300 with the solvents indicated (^1^H NMR at 300 MHz). Chemical shifts are reported in ppm on the δ scale and referenced to the appropriate solvent peak. LCMS were analysed on an Agilent LCMS system equipped with an Agilent G6120B Mass Detector, 1,260 Infinity G1312B Binary pump, 1,260 Infinity G1367E HiPALS autosampler, and 1,260 Infinity G4212B Diode Array Detector. The LCMS conditions were as follows: column: Luna Omega (1.6 µm, C18, 50 × 2.1 mm); injection volume: 1 μl; gradient: 5–100% B over 3.8 min (solvent A: water/0.1% formic acid; solvent B: ACN/0.1% formic acid); acquisition time: 4.1 min; flow rate: 1 ml/min; detection: 254 and 214 nm. Unless otherwise noted, all compounds were found to be >95% pure by this method. The preparative LCMS purification were performed using a Waters preparative HPLC system equipped with Waters ZQ 3100 Mass Detector, Waters 2545-Pump, Waters SFO System Fluidics Organizer, Waters 2,996 Diode Array Detector and Waters 2,767 Sample Manager. The preparative-HPLC conditions were as follows: XBridge BEH C18 OBD Prep Column (130 Å, 5 μm, 19 mm × 100 mm); injection volume: 1 ml; gradient is variable over 20 min depending on each compound (solvent A: water/0.1% formic acid; solvent B: ACN/0.1% formic acid); flow rate, 20 ml/min; detection 100–600 nm. Chiral HPLC analysis was performed with the aforementioned Waters system using a Phenomenex Lux 5u Cellulose-3 (250 × 10 mm) column and a gradient of 45% ACN/55% water at 3 ml/min and 214 nm detection. The specific procedures for the synthesis of analogs of the nematocidal compound—UMW-868—are given in Supplementary File S1.

## Data Availability

The datasets presented in this study has been deposited in the PRIDE repository with the accession number PXD034868.
