# Supplementary material for: Thermal proteome profiling reveals Haemonchus orphan protein HCO_011565 as a target of the nematocidal small molecule UMW-868
Source: Front Pharmacol. 2022 Oct 14;13:1014804. doi: 10.3389/fphar.2022.1014804 (PMC9616048; doi:10.3389/fphar.2022.1014804)
Supplement: Supplementary file 3 [file DataSheet2.docx]

**Supplementary File 1.**

**General Chemistry Procedures.** All non-aqueous reactions were performed under an atmosphere of nitrogen, unless otherwise specified. Commercially available reagents were used without further purification. Flash chromatography was performed with silica gel 60 (particle size 0.040–0.063 μm) on a CombiFlash Rf Purification System (Teledyne Isco) with mobile phase gradients as specified. NMR spectra were recorded on a Bruker Avance DRX 300 with the solvents indicated (^1^H NMR at 300 MHz). Chemical shifts are reported in ppm on the δ scale and referenced to the appropriate solvent peak. LCMS were analyzed on an Agilent LCMS system equipped with an Agilent G6120B Mass Detector, 1260 Infinity G1312B Binary pump, 1260 Infinity G1367E HiPALS autosampler, and 1260 Infinity G4212B Diode Array Detector. The LCMS conditions were as follows: column: Luna Omega (1.6 µm, C18, 50 x 2.1 mm); injection volume: 1 µL; gradient: 5-100% B over 3.8 min (solvent A: water/0.1% formic acid; solvent B: ACN/ 0.1% formic acid); acquisition time: 4.1 min; flow rate: 1 mL/min; detection: 254 and 214 nm. Unless otherwise noted, all compounds were found to be >95% pure by this method. The preparative LCMS purification were performed using a Waters preparative HPLC system equipped with Waters ZQ 3100 –Mass Detector, Waters 2545-Pump, Waters SFO System Fluidics Organizer, Waters 2996 Diode Array Detector and Waters 2767 Sample Manager. The preparative-HPLC conditions were as follows: XBridge BEH C18 OBD Prep Column (130Å, 5 µm, 19 mm X 100 mm); injection volume: 1 mL; gradient is variable over 20 min depending on each compound (solvent A: water/0.1% formic acid; solvent B: ACN/ 0.1% formic acid); flow rate, 20 mL/min; detection 100 to 600 nm. Chiral HPLC analysis was performed with the aforementioned Waters system using a Phenomenex Lux 5u Cellulose-3 (250 x 10 mm) column and a gradient of 45% MeCN/55% water at 3 mL/min and 214 nm detection.

Compounds **A-3126, A-3136, A-6501** and **A-3134** were procured from commercial vendors and used without further purification.

**General procedure A: Preparation of *N*-hydroxy carboximidamide 2**.

To a solution of nitrile **1** (1 eq.) in EtOH (10 mL) was added 50% aq.NH_4_OH solution (5 eq.). The reaction solution was heated to reflux for 4-12 h (monitored by LCMS). The solvent was removed to give the title compound **2**. The product was of sufficient purity to use in the next reaction without further purification.

**(*Z*)-*N*'-Hydroxythiophene-2-carboximidamide**

General procedure **A** was followed^1^ using thiophene-2-carbonitrile (1000 mg, 9.2 mmol). Yield 95%; clear oil (crystalised on standing). ^1^H NMR (300 MHz, DMSO) δ 9.58 (s, 1H), 7.56 – 7.34 (m, 2H), 7.04 (dd, *J* = 5.1, 3.6 Hz, 1H), 5.91 (s, 2H). LC-MS (ESI) t_R_ 0.37 min, m/z 143.2 (M+H)^+^. The ^1^H-NMR spectrum matches previous reported data.

**(*Z*)-*N*'-Hydroxy-4-methoxybenzimidamide**

General procedure **A** was followed^2^ using 4-methoxybenzonitrile (500 mg, 3.8 mmol). Yield 89%; off-white solid. ^1^H NMR (300 MHz, CDCl_3_) δ 7.59 (d, *J* = 8.8 Hz, 2H), 6.93 (d, *J* = 8.9 Hz, 2H), 4.93 (s, 2H), 3.85 (s, 3H). LC-MS (ESI) t_R_ 0.40 min, m/z 167.2 (M+H)^+^.The ^1^H-NMR spectrum matches previous reported data.

**(*Z*)-*N*'-Hydroxy-4-(trifluoromethyl)benzimidamide**

General procedure **A** was followed^3^ using 4-(trifluoromethyl)benzonitrile (500 mg, 2.9 mmol). Yield 93%; off-white solid. ^1^H NMR (300 MHz, DMSO) δ 9.93 (s, 1H), 7.96 – 7.87 (m, 2H), 7.75 (d, *J* = 8.4 Hz, 2H), 5.99 (s, 2H). LC-MS (ESI) t_R_ 0.87 min, m/z 205.2 (M+H)^+^. The ^1^H-NMR spectrum matches previous reported data.

**(*Z*)-*N*'-Hydroxybenzimidamide**

General procedure **A** was followed^4^ using benzonitrile (500 mg, 4.8 mmol). Yield 78%; beige solid. ^1^H NMR (300 MHz, CDCl_3_) δ 7.71 – 7.61 (m, 2H), 7.51 – 7.37 (m, 3H), 4.97 (s, 2H). LC-MS (ESI) t_R_ 0.33 min, m/z 137.2 (M+H)^+^. The ^1^H-NMR spectrum matches previous reported data.

**General procedure B: Synthesis of 1,2,4-oxadiazole 4.**

A mixture of acid **3** (1 eq.), HATU (1.2 eq.) and DIPEA (2.5 eq.) in DMF (1.5 mL) was stirred at r.t for 30 min. To this solution, *N*-hydroxy carboximidamide **2** (1.1 eq.) was added. The reaction solution was heated to 100^o^C 2-12 h, and the solvent was removed. The crude product was subjected to flash column chromatography, eluting with 0-15% EtOAc/heptane to give the title compound **4**.

**5-(4-Propylphenyl)-3-(thiophen-2-yl)-1,2,4-oxadiazole, SP0.**

General procedure **B** was followed using 4-propylbenzoic acid (50 mg, 0.30 mmol) and *N*-hydroxythiophene-2-carboxamidine (48 mg, 0.33 mmol). Yield 55%; colourless solid. ^1^H NMR (300 MHz, CDCl_3_) δ 8.17 – 8.10 (m, 2H), 7.90 (dd, *J* = 3.7, 1.2 Hz, 1H), 7.55 (dd, *J* = 5.0, 1.2 Hz, 1H), 7.42 – 7.34 (m, 2H), 7.21 (dd, *J* = 5.0, 3.7 Hz, 1H), 2.71 (t, *J* = 8.5, 6.8 Hz, 2H), 1.81 – 1.64 (m, 2H), 1.00 (t, *J* = 7.3 Hz, 3H). ^13^C NMR (75 MHz, CDCl_3_) δ 176.0, 165.1, 148.5, 129.7, 129.4, 129.3, 128.8, 128.4, 128.1, 121.7, 38.3, 24.4, 13.9. LC-MS (ESI) t_R_ 3.26 min, m/z 271.2 (M+H)^+^.

**3-(4-Methoxyphenyl)-5-(4-propylphenyl)-1,2,4-oxadiazole, A-6318.**

General procedure **B** was followed using 4-propylbenzoic acid (50 mg, 0.30 mmol) and *N*-hydroxy-4-methoxy-benzamidine (61 mg, 0.37 mmol). Yield 48%; colourless solid. ^1^H NMR (300 MHz, CDCl_3_) δ 8.16 – 8.07 (m, 4H), 7.39 – 7.30 (m, 2H), 7.06 – 6.97 (m, 2H), 3.88 (s, 3H), 2.77 – 2.62 (m, 2H), 1.77 – 1.62 (m, 2H), 0.97 (t, *J* = 7.3 Hz, 3H). ^13^C NMR (75 MHz, CDCl_3_) δ 175.8, 168.7, 162.0, 148.2, 129.3, 129.3, 128.3, 122.1, 119.7, 114.4, 55.5, 38.3, 24.4, 13.9. LC-MS (ESI) t_R_ 3.40 min, m/z 295.2 (M+H)^+^.

**5-Phenyl-3-(thiophen-2-yl)-1,2,4-oxadiazole, A-4347.**

General procedure **B** was followed^5^ using ﻿benzoic acid (50 mg, 0.41 mmol) and *N*-hydroxythiophene-2-carboxamidine (64 mg, 0.45 mmol). Yield 75%; off-white solid. ^1^H NMR (300 MHz, CDCl_3_) δ 8.26 – 8.17 (m, 2H), 7.89 (dd, *J* = 3.7, 1.2 Hz, 1H), 7.67 – 7.50 (m, 4H), 7.19 (dd, *J* = 5.0, 3.7 Hz, 1H). LC-MS (ESI) t_R_ 2.71 min, m/z 229.2 (M+H)^+^. The ^1^H-NMR spectrum matches previous reported data.

**5-(4-(Difluoromethoxy)phenyl)-3-(thiophen-2-yl)-1,2,4-oxadiazole, A-4340.**

General procedure **B** was followed using ﻿4-(difluoromethoxy)benzoic acid (72 mg, 0.39 mmol) and *N*-hydroxythiophene-2-carboxamidine (50 mg, 0.35 mmol). Yield 74%; off-white solid. ^1^H NMR (300 MHz, CDCl_3_) δ 8.30 – 8.20 (m, 2H), 7.90 (dd, *J* = 3.7, 1.2 Hz, 1H), 7.56 (dd, *J* = 5.0, 1.2 Hz, 1H), 7.35 – 7.28 (m, 2H), 7.21 (dd, *J* = 5.0, 3.7 Hz, 1H), 6.66 (t, *J*_H-F_ = 72.9 Hz, 1H). ^13^C NMR (75 MHz, CDCl_3_) δ 174.7, 165.1, 154.5, 130.2, 129.7, 129.4, 128.4, 128.0, 121.0, 119.6, 115.3 (t, *J*_C-F_ = 261.9 Hz). ^19^F NMR (282 MHz, CDCl_3_) δ -81.8. LC-MS (ESI) t_R_ 2.78 min, m/z 295.2 (M+H)^+^.

**3-(Thiophen-2-yl)-5-(4-(trifluoromethoxy)phenyl)-1,2,4-oxadiazole, A-6316.**

General procedure **B** was followed using ﻿4-(trifluoromethoxy)benzoic acid (50 mg, 0.24 mmol) and *N*-hydroxythiophene-2-carboxamidine (37.9 mg, 0.27 mmol). Yield 75%; off-white solid. ^1^H NMR (300 MHz, CDCl_3_) δ 8.30 – 8.20 (m, 2H), 7.88 (dd, *J* = 3.7, 1.2 Hz, 1H), 7.54 (dd, *J* = 5.0, 1.2 Hz, 1H), 7.44 – 7.35 (m, 2H), 7.19 (dd, *J* = 5.0, 3.7 Hz, 1H). ^19^F NMR (282 MHz, CDCl_3_) δ -57.6. ^13^C NMR (75 MHz, CDCl_3_) δ 174.4, 165.2, 152.5, 130.2, 129.8, 129.5, 128.3, 128.1, 122.5, 121.2. LC-MS (ESI) t_R_ 3.07 min, m/z 313.2 (M+H)^+^.

**3-Phenyl-5-(thiophen-2-yl)-1,2,4-oxadiazole, Tioxazafen, A-8417.**

General procedure **B** was followed^6^ using ﻿thiophene-2-carboxylic acid (100 mg, 0.78 mmol) and *N*-hydroxybenzamidine (130 mg, 0.94 mmol). Yield 61%; off-white solid. ^1^H NMR (300 MHz, CDCl_3_) δ 8.19 – 8.11 (m, 2H), 7.97 (dd, *J* = 3.8, 1.2 Hz, 1H), 7.67 (dd, *J* = 5.0, 1.2 Hz, 1H), 7.56 – 7.47 (m, 3H), 7.23 (dd, *J* = 5.0, 3.8 Hz, 1H). LC-MS (ESI) t_R_ 2.73 min, m/z 229.0 (M+H)^+^. The ^1^H-NMR spectrum matches previous reported data.

**5-(4-(Difluoromethoxy)phenyl)-3-(4-(trifluoromethyl)phenyl)-1,2,4-oxadiazole, A-6325.**

General procedure **B** was followed using 4-(difluoromethoxy)benzoic acid (100 mg, 0.53 mmol) and *N*-hydroxy-4-(trifluoromethyl)benzamidine (120 mg, 0.58 mmol). Yield 75%; off-white solid. ^1^H NMR (300 MHz, CDCl_3_) δ 8.37 – 8.22 (m, 4H), 7.80 (d, *J* = 8.2 Hz, 2H), 7.38 – 7.30 (m, 2H), 6.67 (t, *J*_H-F_ = 72.8 Hz, 1H). ^13^C NMR (75 MHz, CDCl_3_) δ 175.2, 168.0, 154.6, 133.0 (q, *J*_C-F_ = 32.6 Hz), 130.2, 127.9, 125.9 (q, *J*_C-F_ = 3.8 Hz), 125.6, 122.0, 121.0, 119.7, 115.3 (t, *J*_C-F_ = 262.2 Hz). ^19^F NMR (282 MHz, CDCl_3_) δ -63.0, -81.9. LC-MS (ESI) t_R_ 3.21 min, m/z 357.2 (M+H)^+^.

**References**

1. Flipo, M. et al. Ethionamide boosters: synthesis, biological activity, and structure− activity relationships of a series of 1, 2, 4-oxadiazole EthR inhibitors. *J. Med. Chem.* **54**, 2994–3010 (2011).
2. Sinha, S., Sravanthi, T. V., Yuvaraj, S., Manju, S. L. & Doble, M. 2-Amino-4-aryl thiazole: a promising scaffold identified as a potent 5-LOX inhibitor. *R.S.C. Adv.* **6**, 19271–19279 (2016).
3. Mayer, J. C. et al. Ferrocenylethenyl-substituted 1, 3, 4-oxadiazolyl-1, 2, 4-oxadiazoles: Synthesis, characterization and DNA-binding assays. *J. Organomet. Chem.* **841**, 1–11 (2017).
4. Lacbay, C. M., Menni, M., Bernatchez, J. A., Götte, M. & Tsantrizos, Y. S. Pharmacophore requirements for HIV-1 reverse transcriptase inhibitors that selectively “Freeze” the pre-translocated complex during the polymerization catalytic cycle. *Bioorg. Chem.* **26**, 1713–1726 (2018).
5. Parker, P. D. & Pierce, J. G. Synthesis of 1, 2, 4-oxadiazoles via DDQ-mediated oxidative cyclization of amidoximes. *Synthesis* **48**, 1902–1909 (2016).
6. Wang, W. et al. Base-mediated one-pot synthesis of 1, 2, 4-oxadiazoles from nitriles, aldehydes and hydroxylamine hydrochloride without addition of extra oxidant. *Org. Biomol. Chem.* **14**, 9814–9822 (2016).
