# Supplementary material for: Thermal proteome profiling reveals Haemonchus orphan protein HCO_011565 as a target of the nematocidal small molecule UMW-868
Source: Front Pharmacol. 2022 Oct 14;13:1014804. doi: 10.3389/fphar.2022.1014804 (PMC9616048; doi:10.3389/fphar.2022.1014804)
Supplement: Supplementary file 4 [file DataSheet1.docx]

Supplementary Material

Thermal proteome profiling reveals *Haemonchus* orphan protein HCO_011565 as a target of the nematocidal small molecule UMW-868

Aya C. Taki^1†^, Tao Wang^1†^, Nghi N. Nguyen^2^, Ching-Seng Ang^3^, Michael G. Leeming^3^, Shuai Nie^3^, Joseph J. Byrne^1^, Neil D. Young^1^, Yuanting Zheng^1^, Guangxu Ma^1,4^, Pasi K. Korhonen^1^, Anson V. Koehler^1^, Nicholas A. Williamson^3^, Andreas Hofmann^1^, Bill C. H. Chang^1^, Cécile Häberli^5,6^, Jennifer Keiser^5,6^, Abdul Jabbar^1^, Brad E. Sleebs^1,2,7^* & Robin B. Gasser^1^*

^1^Department of Veterinary Biosciences, Melbourne Veterinary School, Faculty of Veterinary and Agricultural Sciences, The University of Melbourne, Parkville, Vic 3010, Australia

^2^Walter and Eliza Hall Institute of Medical Research, Parkville, Vic 3052, Australia

^3^Melbourne Mass Spectrometry and Proteomics Facility, The Bio21 Molecular Science and Biotechnology Institute, The University of Melbourne, Parkville, Vic 3010, Australia

^4^Institute of Preventive Veterinary Medicine, Zhejiang Provincial Key Laboratory of Preventive Veterinary Medicine, College of Animal Sciences, Zhejiang University, Hangzhou, Zhejiang, 310058, China

^5^Medical Parasitology and Infection Biology, Swiss Tropical and Public Health Institute, Allschwil, Switzerland

^6^University of Basel, Basel Switzerland

^7^Department of Medical Biology, The University of Melbourne, Parkville, Vic 3010, Australia

*** Correspondence:**Robin B. Gasser and Brad E. Sleebs
robinbg@unimelb.edu.au; sleebs@wehi.edu.au

^†^ These authors contributed equally.

**Supplementary Fig. 1** Dose-response assessment of in vitro activity of hit compound UMW-868 against exsheathed third-stage larvae (xL3s) of *Haemonchus contortus*. The effect of UMW-868 on the larval motility and development to the fourth-stage larva (L4) was assessed to validate activity and establish potency (IC_50_). Two positive controls, monepantel (MON) and moxidectin (MOX), were included as active compound references. Dose-response curves show the inhibition of xL3s of *H. contortus* motility at 90 h (left) and the inhibition of *H. contortus* development at seven days (right). Data points represent three independent experiments conducted in triplicate: the mean ± standard error of the mean (SEM). Images of representative larvae with an *evisceration* (*Evi*) or a *curved* (*Cur*) phenotype are shown.


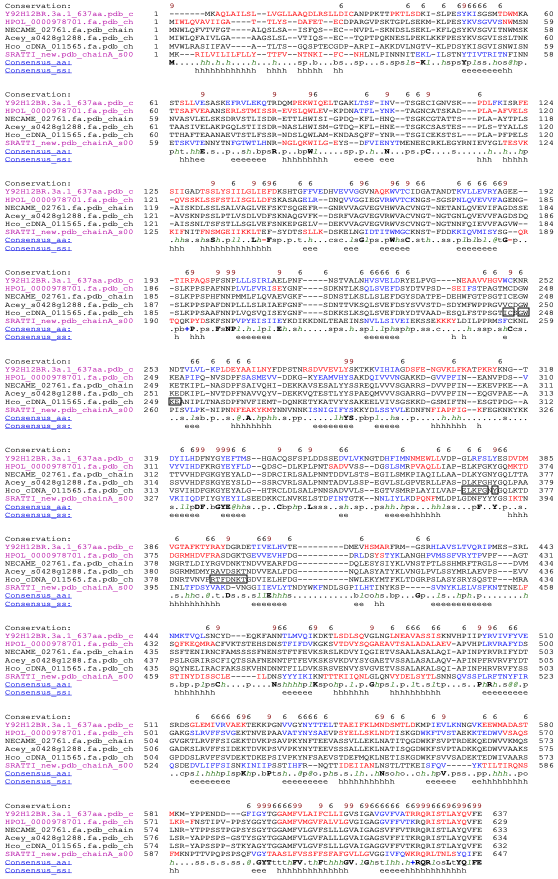


**Supplementary Fig. 2** Tertiary structure-based alignment of HCO_011565 protein orthologs of the nematodes *Caenorhabditis elegans*, *Heligmosomoides polygyrus*, *Necator americanus*, *Ancylostoma ceylanicum*, *Haemonchus contortus* and *Strongyloides ratti* (cf. Supplementary Table 6). The binding sites of HCO_011565 protein (i.e. the residues I244, C245, G247, W248, K249, E250, E365, L366, K367, F368, G369, Y371, R386, T387, F388, D389, N390 and K391) are indicated in black boxes.

**Supplementary Fig. 3** Results from in silico docking of UMW-868 and 10 analogs (Table 1) to protein HCO_011565 of *Haemonchus contortus*. All 10 analogs docked into the same pocket as UMW-868 (Vina scores shown in parentheses; cf. Supplementary Table 6).

**
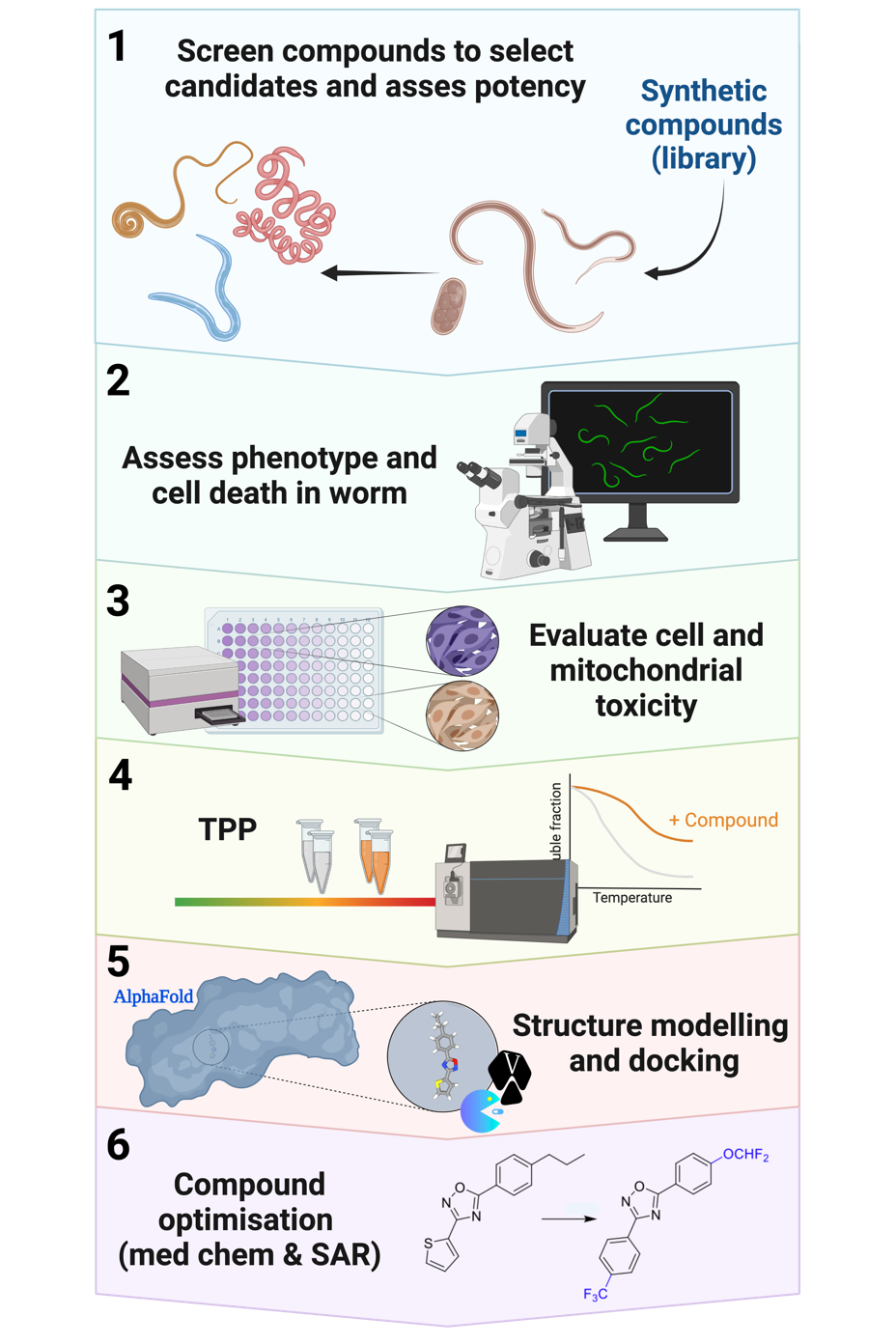
**

**Supplementary Fig. 4.** Summary of the final workflow established in the present study. First, compounds are screened for in vitro activity on larvae, adults and eggs of *Haemonchus contortus* and then on other parasitic nematodes (including *Heligmosmoides polygyrus*, *Necator americanus* and *Trichuris muris*). Second, the phenotype(s) and cell lethality induced in different developmental stages of *H. contortus* and/or other nematode species by the candidate compound(s) are assessed. Third, the toxicities of the candidate compound(s) on cells (cytotoxicity) and mitochondria (mitotoxicity) are assessed in HepG2 human hepatoma cells. Fourth, the method of thermal protein profiling (TPP) is used to explore compound–target interactions and infer target(s). Fifth, molecular docking (using AutoDock) of individual candidate compounds into their potential receptor(s) or binding site(s) within the TPP-inferred target, modelled using the program AlphaFold2. Sixth, the activity and potency of compounds are optimised by medicinal chemistry using structure activity relationship (SAR) investigation(s). The Materials and Methods section provides a detailed description.

**Supplementary Table 1** The 36 ‘hit’ compounds identified in the high throughput screen on *Haemonchus contortus* in the transition from exsheathed third-stage larvae (xL3) to the fourth-stage larvae (L4). Information regarding the compounds (code, chemical name, SMILES and chemical structure), the motility inhibition (≥ 70%) of xL3s after 90 h of incubation and the larval development inhibition to L4 after 168 h of incubation with the test compound (at 20 µM). Abnormal morphological phenotypes observed were curved (*Cur*), evisceration (*Evi*), straight (*Str*), skinny (*Skn*) and coiled (*Coi*). nd = not detected.

| Compound code | Motility reduction  at 90 h (%) | Development inhibition  at 168 h (%) | Abnormal phenotype detected  at 168 h | Chemical name | SMILES | Structure |
| --- | --- | --- | --- | --- | --- | --- |
| MBHF-hit1 | 101.5 | 100 | *Cur* | 2-{2-[4-(trifluoromethyl)phenyl]hydrazono}malononitrile | C(C1=CC=C(NN=C(C#N)C#N)C=C1)(F)(F)F |  |
| MBHF-hit2 | 98.4 | 100 | *Cur, Evi* | 3-[5-(2-phenyleth-1-ynyl)-2-thienyl]-1H-pyrazole | C=1(SC(C#CC2=CC=CC=C2)=CC1)C3=NNC=C3 |  |
| MBHF-hit3 | 96.4 | < 80 | *Cur, Evi* | 1-(2-thienyl)-1-ethanone O-(3-phenyl-1,2,4-thiadiazol-5-yl)oxime | CC(=NOc1nc(ns1)c2ccccc2)c3cccs3 |  |
| MBHF-hit4 | 95.6 | 100 | *Cur, Evi* | 4'-butoxy[1,1'-biphenyl]-4-carbonitrile | CCCCOc1ccc(cc1)c2ccc(C#N)cc2 |  |
| MBHF-hit5 | 95.2 | 100 | *Cur* | 3-(4-chlorophenyl)-2-[2-(2,4-dichlorophenyl)hydrazono]-3-oxopropanenitrile | C(=N/NC=1C(=CC(=CC1)Cl)Cl)(\C(C=2C=CC(=CC2)Cl)=O)/C#N |  |
| MBHF-hit6 | 94.0 | 100 | *Cur, Evi* | 5-[4-(2-phenyleth-1-ynyl)phenyl]-1H-pyrazole | C(#CC1=CC=CC=C1)C=2C=CC(C=3NN=CC3)=CC2 |  |
| MBHF-hit7 | 91.9 | 100 | *Cur, Evi* | 2-(4-chlorophenyl)-3-(methylthio)-1H-indole | C=1(NC=2C=CC=CC2C1SC)C=3C=CC(=CC3)Cl |  |
| MBHF-hit8 | 91.0 | < 80 | *Skn* | 3,4,5,6-tetrachlorophthalonitrile | Clc1c(Cl)c(Cl)c(C#N)c(C#N)c1Cl |  |
| MBHF-hit9 | 89.4 | 100 | *Str* | 3-[(4-chlorophenyl)thio]-1-(2-thienyl)prop-2-en-1-one | C=1(C(/C=C/SC2=CC=C(C=C2)Cl)=O)SC=CC1 |  |
| MBHF-hit10 | 84.2 | < 80 | nd | [3,5-bis(trifluoromethyl)phenyl][4-(4-chlorophenyl)-4-hydroxypiperidino]methanone | OC1(CCN(CC1)C(=O)c2cc(cc(c2)C(F)(F)F)C(F)(F)F)c3ccc(Cl)cc3 |  |
| MBHF-hit11 | 82.7 | < 80 | nd | (2-aminophenyl)[4-(5-nitro-2-pyridyl)piperazino]methanone | [N+](C=1C=NC(N2CCN(C(C=3C(N)=CC=CC3)=O)CC2)=CC1)([O-])=O |  |
| MBHF-hit12 | 82.3 | 80–99 | *Cur, Evi* | N-(4-fluorophenyl)-N'-[1-(4-nitrophenyl)-5-(trifluoromethyl)-1H-pyrazol-4-yl]urea | C1(=C(C=NN1C=2C=CC([N+]([O-])=O)=CC2)NC(NC=3C=CC(=CC3)F)=O)C(F)(F)F |  |
| MBHF-hit13 | 78.8 | < 80 | *Cur* | 3-(2-chlorophenyl)-2-[3-(2-chlorophenyl)-4-methyl-1,3-thiazol-2(3H)-ylidene]-3-oxopropanenitrile | CC1=CSC(=C(C#N)C(=O)c2ccccc2Cl)N1c3ccccc3Cl |  |
| MBHF-hit14 | 81.6 | < 80 | nd | N'-(benzoyloxy)-3-[5-methyl-2-oxo-1,3-benzoxazol-3(2H)-yl]propanimidamide | Cc1ccc2oc(=O)n(CCC(=NOC(=O)c3ccccc3)N)c2c1 |  |
| MBHF-hit15 | 78.7 | < 80 | nd | 1-{[5-(3,5-dichlorophenoxy)-2-furyl]carbonyl}-4-piperidinecarboxamide | C=1(C(N2CCC(C(=O)N)CC2)=O)OC(OC=3C=C(Cl)C=C(C3)Cl)=CC1 |  |
| MBHF-hit16 | 78.5 | 80–99 | *Cur, Evi* | 5-[(4-methoxyphenyl)thio]-3-{[(4-methoxyphenyl)thio]methyl}-1,2,4-thiadiazole | COc1ccc(SCc2nsc(Sc3ccc(OC)cc3)n2)cc1 |  |
| MBHF-hit17 (UMW-868) | 78.4 | 80–99 | *Cur, Evi* | 5-(4-propylphenyl)-3-(2-thienyl)-1,2,4-oxadiazole | CCCc1ccc(cc1)c2nc(no2)c3cccs3 |  |
| MBHF-hit18 | 77.1 | < 80 | *Skn* | 3-oxo-3-[2-(3-pyridyl)-1,3-thiazol-4-yl]-2-{2-[3-(trifluoromethyl)phenyl]hydrazono}propanenitrile | FC(F)(F)c1cccc(NN=C(C#N)C(=O)c2csc(n2)c3cccnc3)c1 |  |
| MBHF-hit19 | 76.9 | < 80 | *Coi* | 4-(5-propyl-2-pyridyl)benzonitrile | CCCc1ccc(nc1)c2ccc(C#N)cc2 |  |
| MBHF-hit20 | 76.7 | 100 | *Str, Cur* | (2,4-difluorophenyl)[5-(2-thienyl)-2-thienyl]methanone | Fc1ccc(C(=O)c2ccc(s2)c3cccs3)c(F)c1 |  |
| MBHF-hit21 | 76.5 | 100 | *Cur, Evi* | 2-(2-{[3-(trifluoromethyl)benzyl]thio}ethyl)-1H-isoindole-1,3(2H)-dione | FC(F)(F)c1cccc(CSCCN2C(=O)c3ccccc3C2=O)c1 |  |
| MBHF-hit22 | 76.3 | < 80 | nd | 3a,6a-dimethylperhydroimidazo[4,5-d]imidazole-2,5-dione | CC12NC(=O)NC2(C)NC(=O)N1 |  |
| MBHF-hit23 | 74.8 | < 80 | nd | N-(2,6-dibromo-4-isopropylphenyl)-N'-(2-furylmethyl)urea | C1(=C(C=C(C=C1Br)C(C)C)Br)NC(NCC=2OC=CC2)=O |  |
| MBHF-hit24 | 74.6 | < 80 | nd | 6-methyl-1-phenylpyrazolo[3,4-d][1,3]oxazin-4(1H)-one | Cc1nc2n(ncc2c(=O)o1)c3ccccc3 |  |
| MBHF-hit25 | 74.5 | 100 | *Cur, Evi* | 4-(5-pentylpyrimidin-2-yl)benzonitrile | CCCCCc1cnc(nc1)c2ccc(C#N)cc2 |  |
| MBHF-hit26 | 73.9 | 80–99 | *Cur, Evi* | 7-chloro-4-(4-chlorophenoxy)quinoline | Clc1ccc(Oc2ccnc3cc(Cl)ccc23)cc1 |  |
| MBHF-hit27 | 73.4 | < 80 | *Cur, Evi* | 5-chloro-N-(4-chlorobenzyl)-4-methoxy-3-thiophenecarboxamide | C=1(C(=C(Cl)SC1)OC)C(NCC=2C=CC(=CC2)Cl)=O |  |
| MBHF-hit28 | 72.7 | < 80 | nd | N-(4-acetylphenyl)-2-{[5-(2-furyl)-4-methyl-4H-1,2,4-triazol-3-yl]sulfanyl}acetamide | CC(=O)c1ccc(NC(=O)CSc2nnc(c3ccco3)n2C)cc1 |  |
| MBHF-hit29 | 72.0 | < 80 | nd | 6-(methylsulfonyl)-1H-indol-1-ol | CS(=O)(=O)c1ccc2ccn(O)c2c1 |  |
| MBHF-hit30 | 71.5 | < 80 | nd | 3-chloro-2,5-dihydro-1H-1lambda~6~-thiophene-1,1-dione | ClC1=CCS(=O)(=O)C1 |  |
| MBHF-hit31 | 71.5 | < 80 | nd | 5-nitro-1-(phenylsulfonyl)indoline | O=N(=O)c1ccc2N(CCc2c1)S(=O)(=O)c3ccccc3 |  |
| MBHF-hit32 | 71.1 | < 80 | nd | 2-[4-(benzyloxy)phenethyl]-1,3-dioxolane | C(Cc1ccc(OCc2ccccc2)cc1)C3OCCO3 |  |
| MBHF-hit33 | 70.3 | 80–99 | *Cur, Evi* | 5-[4-({[5-(trifluoromethyl)-2-pyridyl]thio}methyl)phenyl]-1,2,4-thiadiazole | FC(F)(F)c1ccc(SCc2ccc(cc2)c3ncns3)nc1 |  |
| MBHF-hit34 | 70.3 | < 80 | nd | N1-[(dimethylamino)(methylthio)methylidene]-4-methylbenzene-1-sulfonamide | S(/N=C(/N(C)C)\SC)(C=1C=CC(=CC1)C)(=O)=O |  |
| MBHF-hit35 | 70.2 | < 80 | nd | 1-phenyl-2-[(2-{[4-(trifluoromethyl)pyrimidin-2-yl]amino}ethyl)amino]ethan-1-ol | OC(CNCCNc1nccc(n1)C(F)(F)F)c2ccccc2 |  |
| MBHF-hit36 | 70.2 | < 80 | nd | ethyl 4-(2,4-dichlorophenoxy)-3-nitrobenzoate | CCOC(=O)c1ccc(Oc2ccc(Cl)cc2Cl)c(c1)N(=O)=O |  |

**Supplementary Table 2** Assessment of in vitro activity of hit compound UMW-868 against adult females of *Haemonchus contortus*. The effect of UMW-868 on the motility was assessed over 24 h. Monepantel (MON) and moxidectin (MOX) were included as two positive-reference control compounds. Motility reduction was calculated as mean of triplicates, and given in a percentage (standard error of the mean, SEM).

| **Compound** | **Percentage motility reduction (SEM)** | | | | | |
| --- | --- | --- | --- | --- | --- | --- |
|  | 0 h | 1 h | 2 h | 3 h | 5 h | 24 h |
| UMW-868­ | 0 (0) | 0 (0) | 0 (0) | 0 (0) | 37.5 (12.5) | 66.7 (16.7) |
| MON | 0 (0) | 22.2 (11.1) | 22.2 (11.1) | 25.0 (0) | 42.9 (14.3) | 100 (0) |
| MOX | 0 (0) | 66.7 (0) | 66.7 (0) | 75.0 (12.5) | 71.4 (14.3) | 71.4 (14.3) |
| DMSO (negative control) | 0 (0) | 0 (0) | 0 (0) | 0 (0) | 0 (12.5) | 0 (0) |

**Supplementary Table 3** Assessment of in vitro activity of hit compound UMW-868 against different stages of four species of parasitic nematodes. Lethality in larvae and/or adults exposed to 1 µM (72 h) or 10 µM (40 h) of UMW-868. Mean value of triplicate (standard deviation, SD).

| **Species** | **Development stage** | **Percentage of worms killed (SD)** | |
| --- | --- | --- | --- |
|  |  | **1 µM** | **10 µM** |
| *Ancylostoma ceylanicum* | Third-stage larvae (L3s) | 0 (0) | 93.9 (3.2) |
| *Ancylostoma ceylanicum* | Adults | 50.0 (0) | 100 (0) |
| *Necator americanus* | L3s | Not assessed | 13.4 (4.0) |
| *Heligmosomoides polygyrus* | L3s | Not assessed | 13.0 (2.6) |
| *Strongyloides ratti* | L3s | 31 (4.7) | 100 (0) |
| *Strongyloides ratti* | Adults | Not assessed | 43.9 (7.0) |
| *Trichuris muris* | Adults | Not assessed | 24.1 (1.9) |
| *Caenorhabditis elegans* | Young adults | 0 | 53.5 |

**Supplementary Table 4** Assessment of in vivo activity of hit compound UMW-868 against the nematodes *Heligmosomoides polygyrus* and *Ancylostoma ceylanicum*. The effect of UMW-868 to remove worms in respective hosts (mice or Syrian hamsters) after 72 h was assessed. Mean value of triplicate (standard deviation, SD).

| **Treatment** | **Nematode species** | **Host  animal** | **Dose (mg/kg)** | **No. of animals treated** | **Mean no.**  **of worms**  **(SD)** | **Reduction in infection intensity (%)** |
| --- | --- | --- | --- | --- | --- | --- |
| UMW-868­ | *He. polygyrus* | Mouse | 200 | 4/4 | 12.0 (3.5) | 47 |
| Placebo | *He. polygyrus* | Mouse | 0 | 0/4 | 25.3 (3.9) | 0 |
| UMW-868­ | *A. ceylanicum* | Hamster | 100 | 4/4 | 4.8 (1.0) | 48 |
| Placebo | *A. ceylanicum* | Hamster | 0 | 0/3 | 10 (1.7) | 0 |

**Supplementary Table 5** Full list of *Haemonchus contortus* proteins identified by thermal proteome profiling. (Excel)

**Supplementary Table 6** Supplementary in silico structural and docking raw data. (Excel)
